# Self-adaptive rotational electromagnetic energy generation as an alternative to triboelectric and piezoelectric transductions

**DOI:** 10.1038/s44172-024-00249-6

**Published:** 2024-07-31

**Authors:** Pedro Rolo, João V. Vidal, Andrei L. Kholkin, Marco P. Soares dos Santos

**Affiliations:** 1 Department of Mechanical Engineering and TEMA – Centre for Mechanical Technology & Automation, University of Aveiro, 3810-193 Aveiro, Portugal; 2 Department of Physics and CICECO – Aveiro Institute of Materials, University of Aveiro, 3810-193 Aveiro, Portugal; 3 Department of Physics and I3N, University of Aveiro, 3810-193 Aveiro, Portugal; 4 LASI – Intelligent Systems Associate Laboratory, 4800-058 Guimarães, Portugal

**Keywords:** Energy harvesting, Electrical and electronic engineering

## Abstract

Triboelectric and piezoelectric energy harvesters can hardly power most microelectronic systems. Rotational electromagnetic harvesters are very promising alternatives, but their performance is highly dependent on the varying mechanical sources. This study presents an innovative approach to significantly increase the performance of rotational harvesters, based on dynamic coil switching strategies for optimization of the coil connection architecture during energy generation. Both analytical and experimental validations of the concept of self-adaptive rotational harvester were carried out. The adaptive harvester was able to provide an average power increase of 63.3% and 79.5% when compared to a non-adaptive 16-coil harvester for harmonic translation and harmonic swaying excitations, respectively, and 83.5% and 87.2% when compared to a non-adaptive 8-coil harvester. The estimated energy conversion efficiency was also enhanced from ~80% to 90%. This study unravels an emerging technological approach to power a wide range of applications that cannot be powered by other vibrationally driven harvesters.

## Abstract (web summary)

Pedro Rolo and colleagues designed a rotational electromagnetic harvester using an adaptive coil-switching architecture. This adaptability significantly enhances the energy efficiency and power density, as demonstrated analytically and experimentally.

## Introduction

An increasing interest in the development of high-performance energy harvesters has been observed all over the world, for both small-scale and large-scale applications^1–4^. Emerging small-scale energy harvesting systems are strongly being researched to power a wide range of advanced microelectronic systems, including self-powered remote sensors and/or micro-actuators^5^, mobile devices^6^, portable and micro-wearable systems^7^, and intracorporeal bioelectronic devices^8–10^. If a high energy demand is not required for standalone operations, battery-free solutions have been suggested^11–14^. In addition to their limited lifespan, malfunctioning risks, and associated maintenance operations, substitution can be even impractical in some cases, such as in bioelectronic intracorporeal medical devices, due to inherent risks related to surgical procedures^15,16^. Effective energy harvesting systems must be able to support long-term powering, mainly due to the increasing number of features required by future technologies, which includes intensive monitoring and triggering operations, as well as short transmission/reception periods and dynamic control processing with complex artificial intelligence algorithms^17–21^.

The triboelectric and piezoelectric harvesters^22–27^ can be designed according to a wide range of architectures^28–30^, and can provide high open-circuit output voltages (that can exceed 1 kV) when triggered by mechanical vibrations, a ubiquitous source readily available in nature, industry and transportation systems, etc^20^. Nevertheless, as they are low current sources with high parallel internal impedance, they are only able to provide electric currents in the nano-micro range^31–37^, which can hardly power most microelectronic systems, mainly if energy mechanical sources are intermittent, and advanced processing and communication capabilities are required^20,21,38–41^. Included are the recent works published by An et al.^42^ Abdelkareem et al.^43^ and Liang et al.^44^ which reported peak electric currents usually <200 ${{{\rm{\mu }}}}{{{\rm{A}}}}$, even for multidirectional mechanical excitation dynamics. Under this scenario, complex signal conditioning and energy storage systems are required and energy production costs are higher^40,41,45–48^. A strong research trend aiming to solve these problems is focused on the design of hybrid harvesters, in particular triboelectric/piezoelectric-electromagnetic harvesters^36,49^, even though some designs also include the three transductions mechanisms (triboelectric-piezoelectric-electromagnetic harvesters) to operate simultaneously^50,51^. Differently, electromagnetic transduction mechanisms are typically low voltage sources and can also be readily used to provide electric currents >1000-fold higher than triboelectric/piezoelectric harvesters^36^. The relevant results provided by Peng et al.^52^ Rahman et al.^53^ and Zhou et al.^54^ highlight electric currents that can exceed 100 mA. Electromagnetic harvesters (EMGs) can be designed using different architectures, usually using coils and/or magnets incorporated into distinct co-moving parts^36^. Recent breakthroughs permit that high performance, simple design, and low manufacturing/maintenance costs can co-exist^46,55^. EMGs have geometric architectures that can be broadly characterized as: (i) linear^47,56^; (ii) rotational^55,57–61^ or (iii) multidimensional^50,62,63^. Rotational harvesters (RHs) in particular can combine components for low-friction axial motion dynamics, such as spherical bearings, ensuring higher energy efficiency and durability when compared to other architectures^36^. Notice that, although triboelectric-piezoelectric hybridization has been considered a promising methodology, they are not still able to provide long-term, robust, and reliable operation^64–66^. Besides, the periodic arrangement of the coils embedding RHs along the angular direction also yields rotation symmetric architectures, which simplify the physical modelling, as well as internal and external electrical circuitries^67^. Pendulum-based electromagnetic harvesters are sensitive to changes in the spatial orientation of the generator’s body in relation to the gravitational field of Earth, and, thus, they are well directed for applications involving general vibrations^68^. However, current pendulum-based RHs present relatively large resonance frequencies and are not able to maximize energy generation by performance adaptability to external mechanical energy sources^55^. A wide range of technologies, including for small-scale applications (e.g. using human motions) and large-scale applications (e.g. using wind and/or sea waves^40,69–71^) exhibit low frequencies which change with time^40,55,72–74^. Besides, various rotational architectures operate with eccentric masses which^36,55^, depending on its total mass, shape and positioning, results in different frequencies of resonance^57–59^. Maximization of power density has been mainly proposed by geometric matching to *a priori* known excitations with given frequencies and amplitudes^20,21,47^. Nevertheless, this is a widely used methodology that does not consider the significant changes occurring in the mechanical excitation sources, which can significantly reduce the harvesters’ performance^20,21^.

Recently, some very promising self-adaptive linear harvesters have been proposed to change harvester’s characteristics throughout power generation, including the harvester’s length or the number of active coils^20,21^. The use of mobile components^57^ to provide some adaptability degree is relevant, as it reduces the dependence on resonance frequency^75–77^. Even so, energy losses can be significantly minimized by controlling Ohmic losses related to the internal resistance of the coils, as well as the electromechanical coupling coefficient^78^. In this research, we focused on the concept of self-adaptive rotational EMG incorporating a control system to perform two advanced coil switching strategies: (i) turning off coils not contributing to the generated electromotive forces, and (ii) reversing its polarity to avoid electromotive forces from canceling each other. We here demonstrate that this harvester is able to autonomously optimize the switching of electrical connections between the multiple coils, in such a way that the energy conversion efficiency is maximized for all mechanical configurations of the RH and input vibrational mechanical excitations. Our adaptive strategy was able to increase the power output from 5.1 mW to 10 mW and the energy conversion efficiency from $\approx$80% to 90%. This work provides a strong contribution to the design of adaptive strategies for rotational harvesters, even though the underlying concept can be applied to more complex electromagnetic harvesters, including hybrids, in which the combined advantages of both triboelectric/piezoelectric and electromagnetic harvestings can be obtained. This is an impacting engineering solution that holds the potential to be an alternative to both triboelectric and piezoelectric generation, where such minimization of the internal energy losses is not so straightforward.

## Results and Discussion

### Structural design overview

**Fig. 1:**
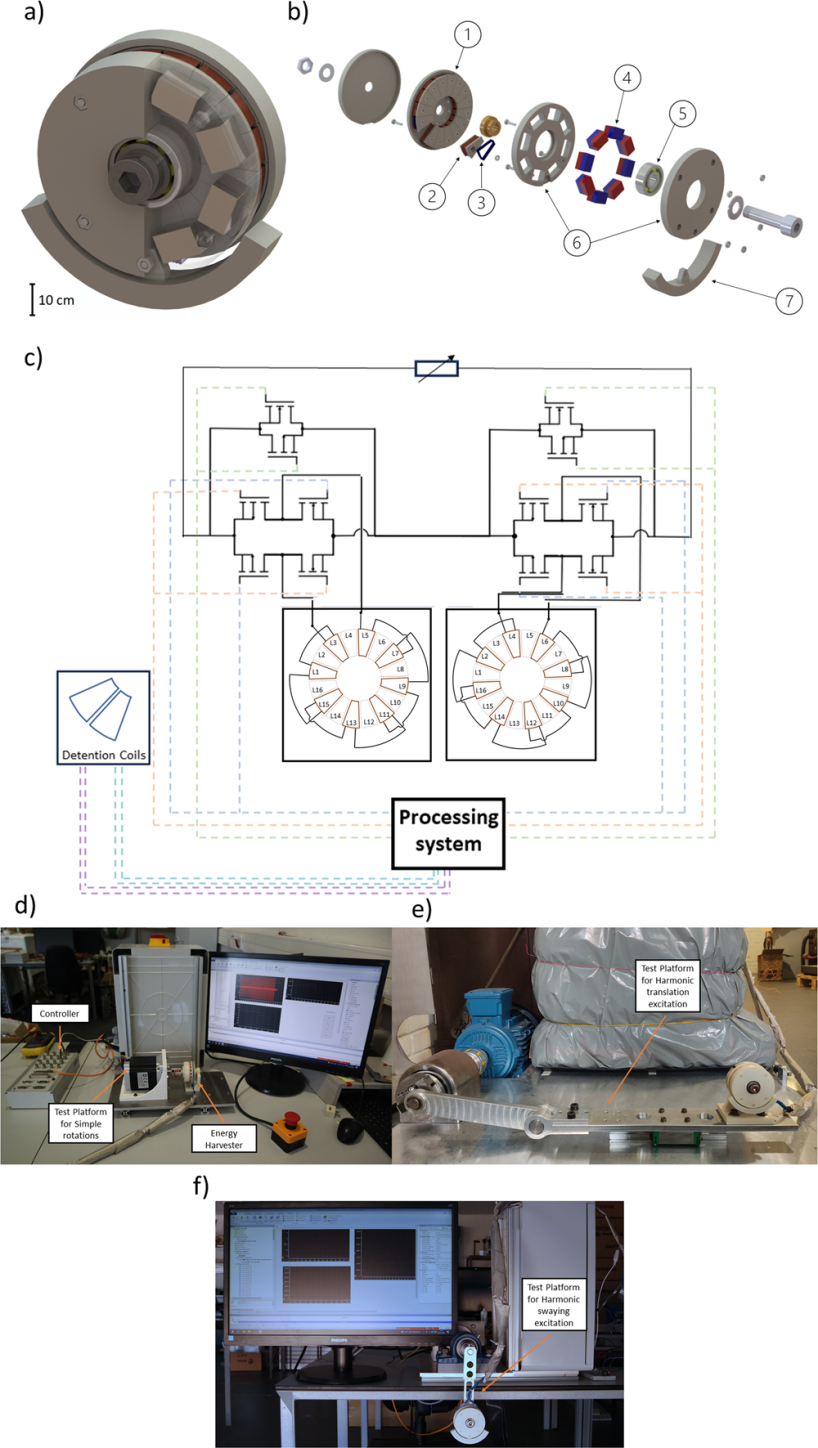
Mechanical and electric components of the adaptive harvesters and testing apparatus. **a** Photo-realistic representation of the developed rotational harvester. **b** Photo-realistic exploded view of the rotational harvester illustrating its different components (1 – stator; 2 – circular sector-shaped single coil fixed to the stator; 3 – overlapping detention pickup coil; 4 – permanent magnets fixed on the rotator; 5 – ceramic ball bearing; 6 – rotator; 7 – eccentric mass). **c** Diagram of the adaptive rotational harvester incorporating its four main internal systems: (i) processing system; (ii) detection pickup coils; (iii) coil group terminal switching system; and (iv) variable resistance. **d** Photo of the experimental apparatus used to apply mechanical axial rotations to the rotational harvester; **e** Apparatus for testing the translational motion with harmonic excitations, and **f**. apparatus for testing the swaying motion with harmonic excitations.

The RH consists of a rotator, a stator, an eccentric mass, and an instrumentation system to perform the coil-switching strategies. As illustrated in Figs. 1a and b, the rotator is composed of eight sets of four stacked magnets fixed to the rotator. Magnets were arranged ensuring that the magnetic polarity of each set of magnets alternates with each other. The stator consists of 16 radially disposed coils and is separated into two distinct groups of 8 even or odd-numbered coils alternately connected in series between themselves. Each group of coils is electrically connected in such a way that terminals with a given electrical polarity are connected to terminals with the same polarity. This harvester was designed to ensure a small axial distance between magnets and coils, such that higher power density and efficiency gains can be obtained. The eccentric mass was included to establish an imbalance in the mass distribution of the harvester, which causes changes both in the center of mass and moment of inertia, making the architecture sensitive to orientation changes relative to Earth’s gravitational field. Finally, instrumentation comprising switching and processing systems was provided (Fig. 1c).

### Features of the adaptive harvester

To maximize the performance of the RH, a ceramic ball bearing was incorporated in the harvester’s geometric center to provide low friction during rotation. Its architecture was developed through rapid prototyping methods in a 3D printer (Ender-3 S1 3D Printer, Creality), using Polylactic Acid (PLA), due to its negligible magnetic properties and electrical conductivity. Both rotor and extractor were designed with 61 mm in diameter, resulting in 72.47 cm^3^ of total volume. 1.8 mm of spacing between the coil-magnets was established. Additionally, an eccentric mass was positioned at one end of the RH to achieve a low resonance frequency. This eccentric mass was designed to weigh 17.24 g and to be uniformly distributed along a volume of 6.3 cm^3^, located concentrically 2 mm from the outer edge of the RH.

### Instrumentation of the adaptive harvester

Our adaptive RH comprises three distinct instrumented systems (Fig. 1c): (i) a monitoring system, to detect the current mechanical configuration of the rotator relative to the stator, as well as its rate of change; (ii) a processing system running a algorithm to implement the coil switching strategies, which includes finding the best electrical configuration of the coils according to the stator-rotator state; (iii) the switching hardware with low power consumption electronics that implement the different coil configurations for optimized RH performance. Two coils were engineered as detection pickup coils, such that the stator-rotator dynamics could be monitored, and power consumption related to the use of passive sensing systems could be eliminated^21^. Those were winded up around two adjacent power coils in the stator. These coils were used to determine the optimum configuration of the power coils through the measured open-circuit voltage, thus producing no additional Lorentz braking forces and associated power losses. As the architecture of our harvester is not complex and experimental tests were carried out at low-frequency, no dynamic controller (e.g. PID control) was used. Instead, a control algorithm was designed based on three if-else rules established to output the switching dynamics according to the stator-rotator dynamics monitored by these detection coils. Taking ${V}_{{th}-n}=100$ ${{{\rm{mV}}}}$ as the voltage induced on the specific detection coil $n$ surpassing an established threshold, ${V}_{c-n}$ the voltage induced on the specific power coil, and considering that each power coil is configured as $+1$, $0,$ or $-1$ (the sign of ${V}_{{th}-n}$ was used to identify which terminals of the power coils must be connected to ensure the superposition of electric currents: $+1$ representing the case with the $+$ and $\mbox{--}$ signs of an output circuit is connected to the $+$ and $\mbox{--}$ signs of the individual coil; $-1$ representing the opposite case; and $0$ representing a disconnected loop; section 3 provides additional details), then,

If ${V}_{c-n} \, > \, {V}_{{th}-n}$, then configuration $+1$ is set;

If $-{V}_{{th}-n}\, \le \,{V}_{c-n} \, < \, {V}_{{th}-n}$, then configuration $0$ is set;

${V}_{c-n} \, < \, -{V}_{{th}-n}$, then configuration $-1$ is set.

When the ${V}_{c-n} \, > \, {V}_{{th}-n}$ or ${V}_{c-n} \, < \,-{V}_{{th}-n}$, the current extractable from the corresponding power coil is added to the current extracted by the whole device, in such a way as to optimize its output power and efficiency while minimizing the Ohmic losses. Although deactivating power coils implies open-circuiting them, short-circuiting power coils will introduce an additional braking to the rotator, due to non-null currents flowing in them, although also avoiding the possibility of inductive voltage spikes and instability of the processing system, since the additional braking from short-circuiting decreases the pick-up open-circuit voltage in a negative feedback loop. The processing operations are carried out by an extremely low power consumption microcontroller (MSP430, Texas Instruments), which requires only 66 µW to perform the processing actions, including those for monitoring and control operations. The adaptive RH system is composed of two subsystems: (1) a coil switching system, which performs on/off switching of coil groups; and (2) a reversing polarity switching, capable of modifying the polarization of these coil groups. The first sub-system is composed of two bilateral transmission gate switches, which consist of two types of MOSFETs, namely NMOS (XPQR3004PB) and PMOS (IRF4905PbF) (Fig. 1c). The second sub-system is implemented using an H-bridge (HT8835A), also composed of MOSFETS, connected to each coil group.

### Analytical model

The operation of the EMG can be explained by a general model developed from first principles relying on the balance laws of mass, linear momentum, angular momentum, and energy as well as the laws of electrodynamics^78^. External contact mechanical tractions applied to the stator/rotator result in corresponding 3D motions and associated inertial forces applied to the rotator/stator. Movement of the incorporated magnets relative to the stator coils thus generates electromotive forces as described by Faraday’s Law of induction. Overall, the device converts the input mechanical power into a time rate of change of kinetic, potential, and inductive energies and outputs useful power in an electrical circuit as well as wasted components in the form of friction and Ohmic losses.

**Fig. 2:**
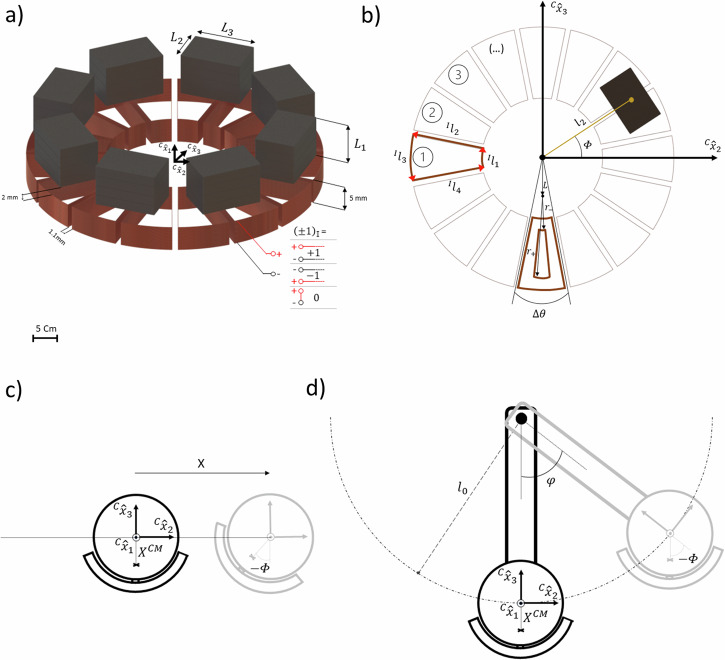
Coil-magnet interface of the adaptive harvester and test excitations. **a** Tridimensional and **b** bidimensional representation of the permanent magnets and coils of the electromagnetic harvester, and corresponding dimensions, as well as the electrical current paths associated with each loop of a coil. **c** Illustration of the electromagnetic harvester subjected to a translational motion and corresponding Cartesian axis, angles, and relevant distances. **d** Illustration of the electromagnetic harvester subjected to a pendular motion and corresponding Cartesian axis, angles, and relevant distances.

As depicted in Figs. 1b and 2a, the system under study can be divided into a: (i) rotator occupying a volume ${\!\,}^{M}v$ and containing 8 permanent magnets, rigid material, and the outer part of the bearing; and (ii) stator occupying a volume ${\!\,}^{C}v$ and containing 16 coils, rigid material, and the inner part of the bearing. We consider an inertial frame with Cartesian coordinates ${x}_{i}$ ($i$ $\in$ $\left\{{{\mathrm{1,2,3}}}\right\}$) as well as two non-inertial frames with origin on the center of symmetry of the stator which respectively follow the motion of the rigid stator and rotator and with corresponding Cartesian coordinates in their respective basis ${{\!\,}^{C}x}_{I}$ and ${{\!\,}^{M}x}_{{I}^{{\prime} }}$ ($I$ $\in$ $\left\{{{\mathrm{1,2,3}}}\right\}$ and ${I}^{{\prime} }$ $\in$ $\left\{{{\mathrm{1,2,3}}}\right\}$) (Fig. 2b,c)^78^. If all of the components are assumed to behave as rigid bodies, the material points in each of these volumes can be described in the inertial frame as (using summation convention):

1 $$\left\{\begin{array}{c}{x}_{i}={T}_{i}+{R}_{{iI}}{\delta }_{I}+{R}_{{iI}}{{R}_{\varPhi }}_{I{I}^{{\prime} }}{\!\,}^{M}X_{{I}^{{\prime} }},\,{{{\boldsymbol{x}}}}\in {\!\,}^{M}v\\ {x}_{i}={T}_{i}+{R}_{{iI}}{\!\,}^{C}X_{I},\,{{{\boldsymbol{x}}}}\, {{\in }}\, {\!\,}^{C}v\end{array}\right.,$$

where ${T}_{i}(t)$ is a time-dependent translation vector of the center of the stator in relation to the origin of the inertial referential, ${R}_{{iI}}(t)$ a time-dependent rotation matrix of the stator and ${{R}_{\varPhi }}_{I{J}^{{\prime} }}(t)$ the time-dependent rotation matrix of the rotator in relation to the stator, both belonging to the 3D rotation group SO(3). ${\delta }_{I}$ is the constant distance between the geometric center of the stator and rotator, ${\scriptstyle{M}\atop }\!X_{I'}$ the time-independent points of the rotator and ${\!\,}^{C}X_{I}$ the points of the stator in respective reference material frames. The rotator is physically constrained to rotations by an angle $\varPhi (t)$ around the axial $I$ = 1 direction and thus the corresponding rotation matrix is:

2 $${{{{\boldsymbol{R}}}}}_{\varPhi }\left(t\right)=\left(\begin{array}{ccc}1 & 0 & 0\\ 0 & \cos \left(\varPhi \right) & -\sin \left(\varPhi \right)\\ 0 & \sin \left(\varPhi \right) & \cos \left(\varPhi \right)\end{array}\right).$$

The EMG therefore only comprises a single mechanical degree of freedom. From the relation: ${\varepsilon }_{{I}^{{\prime} }{J}^{{\prime} }{K}^{{\prime} }}{\omega }_{{J}^{{\prime} }}^{\varPhi }={\left({{{{\boldsymbol{R}}}}}_{\varPhi }^{T}\dot{{{{{\boldsymbol{R}}}}}_{\varPhi }}\right)}_{{I}^{{\prime} }{K}^{{\prime} }}$, the corresponding angular velocity associated with this rotation simply has the axial component: ${\omega }_{{I}^{{\prime} }}^{\varPhi }={\delta }_{{I}^{{\prime} }1}\dot{\varPhi }$, where ${\varepsilon }_{{IJK}}$ is the permutation Levi-Civita symbol and ${\delta }_{{IJ}}$ is the Kronecker delta symbol.

**Fig. 3:**
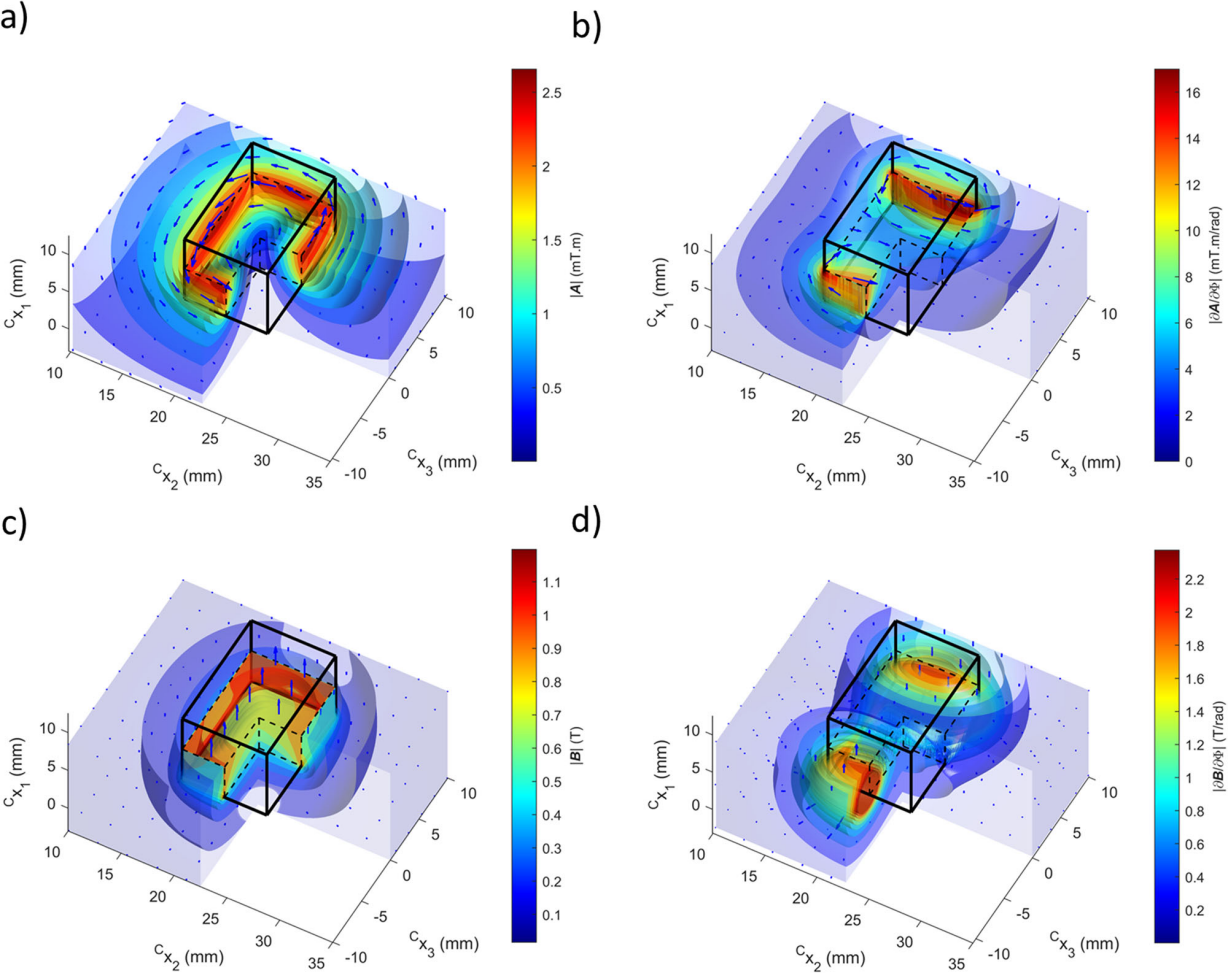
Calculated three-dimensional isosurfaces of the magnetic fields produced by one of the rectangular permanent magnets of the harvester cut along its transverse ^*c*^*x*_1_=*δ*_*1*_ middle plane (with its amplitude represented in color scale and local directions represented by arrows). **a** Magnetic vector potential; **b** Rate of change of the magnetic vector potential with an infinitesimal change in the $\varPhi$ angle of the rotator. **c** Magnetic induction field, and **d** Rate of change of the magnetic induction field with an infinitesimal change in the $\varPhi$ angle of the rotator

A permanent rectangular magnet may be assumed to have a constant magnetization vector pointing in the axial direction and be analytically described by:

3 $${M}_{i}={R}_{{iI}}{{R}_{\varPhi }}_{I{I}^{{\prime} }}{\delta }_{{I}^{{\prime} }1}M,\,{\!\,}^{M}X_{{I}^{{\prime} }}\in {\!\,}^{M}V;$$

$${\!\,}^{M}V=\left\{\left({\!\,}^{M}X_{1},{\!\,}^{M}X_{2},{\!\,}^{M}X_{3}\right){{{\rm{|}}}}l_{{I}^{{\prime} }}-{L}_{{I}^{{\prime} }}/2 \, < \, {\!\,}^{M}X_{{I}^{{\prime} }} \, < \, l_{{I}^{{\prime} }}+{L}_{{I}^{{\prime} }}/2\right\},$$

where $M$ is the amplitude of the magnetization, $l_{{I}^{{\prime} }}$ are the Cartesian components of the geometric center of the magnet in the material basis of the rotator and ${L}_{{I}^{{\prime} }}$ are its lengths in the various directions. From the Maxwell’s equations in the quasi-magnetostatic form, the components of the magnetic vector potential due to such magnet ${\!\,}^{M}{{{\boldsymbol{A}}}}$ may be written in integral form as:

4 $${\,\!}^{M}A_{i}= -\frac{{\mu }_{0}}{4\pi }\int\limits_{\sigma \,}\,\frac{{\varepsilon }_{{ijk}}{n}_{j}^{{\prime} }{M}_{k}}{\left|{{{\boldsymbol{x}}}}-{{{{\boldsymbol{x}}}}}^{{{{\prime} }}}\right|}d{a}^{{\prime} }\\ = \, {R}_{{iI}}{{R}_{\varPhi }}_{I{J}^{{\prime} }}\frac{{\mu }_{0}M}{4\pi }\left(-{\delta }_{{I}^{{\prime} }2}\int\limits _{l_{2}-\frac{{L}_{2}}{2}}^{l_{2}+\frac{{L}_{2}}{2}}\int\limits_{l_{1}-\frac{{L}_{1}}{2}}^{{l_{1}+\frac{{L}_{1}}{2}}}fd{\!\,}^{M}X_{1}d{\!\,}^{M}X_{2} \;\right]_{{\!\,}^{M}X_{3}=l_{3}-\frac{{L}_{3}}{2}}^{{\!\,}^{M}X_{3}=l_{3}+\frac{{L}_{3}}{2}}\\ \left.\left.+{\delta }_{{I}^{{\prime} }3}\int \limits_{l_{3}-\frac{{L}_{3}}{2}}^{l_{3}+\frac{{L}_{3}}{2}}\int\limits_{l_{1}-\frac{{L}_{1}}{2}}^{l_{1}+\frac{{L}_{1}}{2}}fd{\!\,}^{M}X_{1}d{\!\,}^{M}X_{3} \; \right]_{{\!\,}^{M}X_{2}=l_{2}-\frac{{L}_{2}}{2}}^{{\!\,}^{M}X_{2}=l_{2}+\frac{{L}_{2}}{2}}\right){{{\rm{;}}}}$$

$$f=\frac{1}{\sqrt{{\!\,}^{M}X_{{I'}}{\!\,}^{M}X_{{I'}}-2({\!\,}^{C}X_{I}-{\delta }_{I}){{R}_{\varPhi }}_{I{I'}}{\!\,}^{M}X_{{I'}}+({\!\,}^{C}X_{I}-{\delta }_{I})({\!\,}^{C}X_{I}-{\delta }_{I})}},$$

where $\sigma$ is the surface area of the magnet and ${\mu }_{0}$ is the vacuum permeability. The magnetic induction field is given by the rotational of this field: ${\!\,}^{M}{{{\boldsymbol{B}}}}=\nabla \times {\!\,}^{M}{{{\boldsymbol{A}}}}$. Notably, Eq. (4) shows that the associated vector potential only has components perpendicular to the ${I}^{{\prime} }$ = 1 axis of symmetry of the harvester. In order to calculate the magnetic fields depicted in Fig. 3, the first integral was solved algebraically and the remaining one numerically in Matlab. As illustrated in the 3D representation of the permanent magnet of Fig. 3a, the corresponding magnetic vector field in the frame of the stator (${\!\,}^{M}A_{I}={R}_{{Ii}}^{T}{\!\,}^{M}A_{i}$) has non-axial components that point in a counterclockwise direction, as seen from the top, and its magnitude is maximal in the central region along the side walls of the magnet. The corresponding magnetic induction field is plotted in Fig. 3c, showing that the field is much stronger inside the magnet and mostly points in the axial direction, while its magnitude quickly decreases with the distance from the magnet.

A coil in the stator is composed of multiple loops of wire, each one indexed by a ${{{\mathscr{L}}}}\, {{{\mathscr{\in }}}}\, {\mathbb{N}}$ variable, which counts the number of the loop in the axial direction, from the top to the bottom, and a ${{{\mathscr{R}}}}\, {{{\mathscr{\in }}}}\,{\mathbb{N}}$ variable, which counts the number in the radial direction, from the inside into the outside. Each loop has a cylindrical symmetry and its central position may be decomposed into 4 different paths (Fig. 2b). The various paths of a densely packed coil can be analytically described by the sets:

5 $$\left({\!\,}^{C}X_{1},{\!\,}^{C}X_{2},{\!\,}^{C}X_{3}\right)=\left(z,\,r\cos (\theta )-L+\left[{{{\mathscr{R}}}}\, {{{\mathscr{-}}}}\, 1\right]\frac{\sqrt{3}}{2}l,r\sin \left(\theta \right)\right){{{\rm{;}}}}$$

$$l=\frac{d}{\sin \left(\Delta \theta /2\right)};L=\frac{D}{\sin \left(\Delta \theta /2\right)};$$

$${\,\!}^{I}l_{1}=\left\{\left(z,r,\theta \right){|\; z} ={z}_{0}-\left[{{{\mathscr{L}}}}-1\right]d-\frac{1}{4}\left[1+{\left(-1\right)}^{{{{\mathscr{R}}}}}\right]d,r \right.\\ \left. ={r}_{-}+\left[{{{\mathscr{R}}}}-1\right]\left[l-d\right]\frac{\sqrt{3}}{2},\pi -\Delta \theta /2 \, < \, \theta \, < \, \pi +\Delta \theta /2\right\};$$

$${\!\,}^{I}l_{2}= \left\{\left(z,r,\theta \right){|\; z}={z}_{0}-\left[{{{\mathscr{L}}}}-1\right]d-\frac{1}{4}\left[1+{\left(-1\right)}^{{{{\mathscr{R}}}}}\right]d,{r}_{-}\right.\\ \left.+\, \left[{{{\mathscr{R}}}}-1\right]\left[l-d\right]\frac{\sqrt{3}}{2} \, < \, r \, < \, {r}_{+}+\left[{{{\mathscr{R}}}}-1\right]\left[l+d\right]\frac{\sqrt{3}}{2},\theta =\pi -\Delta \theta /2\right\};$$

$${\!\,}^{I}l_{3}= \left\{\left(z,r,\theta \right){|\; z}={z}_{0}-\left[{{{\mathscr{L}}}}-1\right]d-\frac{1}{4}\left[1+{\left(-1\right)}^{{{{\mathscr{R}}}}}\right]d,r={r}_{+}\right.\\ \left.+\, \left[{{{\mathscr{R}}}}-1\right]\left[l+d\right]\frac{\sqrt{3}}{2},\pi -\Delta \theta /2 \, < \, \theta \, < \, \pi +\Delta \theta /2\right\};$$

$${\!\,}^{I}l_{4}= \left\{\left(z,r,\theta \right){|\; z}={z}_{0}-\left[{{{\mathscr{L}}}}-1\right]d-\frac{1}{4}\left[1+{\left(-1\right)}^{{{{\mathscr{R}}}}}\right]d,{r}_{-}\right.\\ \left.+\left[{{{\mathscr{R}}}}-1\right]\left[l-d\right]\frac{\sqrt{3}}{2} \, < \, r \, < \, {r}_{+}+\left[{{{\mathscr{R}}}}-1\right]\left[l+d\right]\frac{\sqrt{3}}{2},\theta =\pi +\Delta \theta /2\right\},$$

where $\Delta \theta$ is the angle spanned by the coil, $d$ is the diameter of the wire and $D$ is a known distance, ${z}_{0}$ is the axial position of the center of the top loop, ${r}_{-}$ is the inner radius and ${r}_{+}$ is the outer radius of the innermost loop. From the law of conservation of charge, a given current must flow between each of these sequential paths in a counterclockwise direction (i.e. with decreasing $\theta$ in ${\!\,}^{I}l_{1}$, increasing $r$ in ${\!\,}^{I}l_{2}$, increasing $\theta$ in ${\!\,}^{I}l_{3}$ and decreasing $r$ in ${\!\,}^{I}l_{4}$). The parameters of the engineered harvester are presented in Table 1. From the Faraday’s law of induction, an electromotive force ($\xi$) is generated in a current loop proportionally to the time change of the magnetic flux (${\varPhi }_{B}$) over a surface $s$ delimited by the path $\partial s$. Approximating the wire as an infinitesimally thin loop transporting a total conductive current $I$ and assuming this loop is not deformed with time results in the electromotive force:

6 $$\xi =-\frac{d}{{dt}}{\varPhi }_{B} = -\frac{d}{{dt}}\int\limits_{s}\,{{{\boldsymbol{B}}}}.\, {{{\boldsymbol{n}}}}{da}=-\frac{d}{{dt}}{\oint }_{\partial s}\,{{{\boldsymbol{A}}}}.\, d{{{\boldsymbol{l}}}} \\ =-\dot{\varPhi }\left(\int\limits_{S}\,\frac{\partial {\!\,}^{\,M}B_{I}}{\partial \varPhi }{N}_{I}{dA}\right)-\dot{I}\left(\int\limits_{S}\,\frac{\partial {\!\,}^{\,I}B_{I}}{\partial \varPhi }{N}_{I}{dA}\right) \\ =-\dot{\varPhi }\left({\oint }_{\partial S}\,\frac{\partial {\!\,}^{\,M}A_{I}}{\partial \varPhi }d{\!\,}^{C}X_{I}\right)-\dot{I}\left({\oint }_{\partial S}\,\frac{\partial {\!\,}^{\,I}A_{I}}{\partial I}d{\!\,}^{C}X_{I}\right),$$

where ${\!\,}^{{I}}B_{I}$ and ${\!\,}^{{I}}A_{I}$ are respectively the magnetic induction and vector fields produced by a current $I$ flowing in the loop. This shows that the open-circuit voltage generated on the loop will be proportional to the derivatives of the magnetic fields relative to the $\varPhi$ angle. Figure 3b and d show the calculated changes in magnetic fields in space with a change in the rotation angle, respectively. The results of the integration of these fields over the closed paths or surfaces of the loops are the electromechanical coupling factors, quantifying the open-circuit voltages induced on such paths for a constant angular velocity of 1 rad s^-1^. Figure 3b and d show that most of the regions of high field change are concentrated next to the two surfaces of the magnet normal to the direction of the motion. They also point in a different direction between the two diametrically opposed surfaces. Thus, it should be clear that a loop that maximizes such an EM factor for this configuration of the system corresponds to one that spans a large are in the ${\!\,}^{C}x_{1}=\,{\delta }_{1}$ plane covering one of these two normal surfaces up to the middle ${\!\,}^{C}x_{3}=\,0$ cross plane. Regardless, since the resistance of the wire increases with its length and correspondingly the Ohmic losses, from the point of view of the output power and efficiency a more useful parameter to maximize is the power factor equal to the square of the aforementioned integral divided by the length of the path. Thus, the optimum loop shape should correspond to one that essentially only covers a small area around the region of high magnetic field change in Fig. 3d, hence explaining the introduction of a higher number of coils than magnets in the design of our EMG.

**Table 1:** Parameters of the adaptive harvester

${{{\boldsymbol{M}}}}$ (MA m^-1^)	${{{\boldsymbol{\delta }}}}$ (mm)	${{{{\boldsymbol{l}}}}}_{{{{\boldsymbol{1}}}}}$ (mm)	${{{{\boldsymbol{l}}}}}_{{{{\boldsymbol{2}}}}}$ (mm)	${{{{\boldsymbol{l}}}}}_{{{{\boldsymbol{3}}}}}$ (mm)	${{{{\boldsymbol{L}}}}}_{{{{\boldsymbol{1}}}}}$ (mm)	${{{{\boldsymbol{L}}}}}_{{{{\boldsymbol{2}}}}}$ (mm)	${{{{\boldsymbol{L}}}}}_{{{{\boldsymbol{3}}}}}$ (mm)	${{{\boldsymbol{m}}}}$ (g)	${{{{\boldsymbol{X}}}}}^{{{{\boldsymbol{CM}}}}}$ (mm)
1.142	8.5	0	22.6	0	8	8	12	88.8	15
${{{\boldsymbol{d}}}}$ (μm)	$D$ (mm)	$\Delta \theta$ (deg.)	${z}_{0}$ (mm)	${r}_{-}$ (mm)	${r}_{+}$ (mm)	${N}_{{{{\mathscr{L}}}}}$	${N}_{{{{\mathscr{R}}}}}$	${R}^{{\mathbb{I}}}\,(\Omega )$	${I}_{1}$ (g.m^2^)
68	2.5	22	2.5	1.9	13.4	73	15	220	0.1376

It includes the magnetization of each magnet ($M$), axial distance between stator and rotator ($\delta$), coordinates of the geometric center of one of the rectangular magnets (${l}_{1},{l}_{2},{l}_{3}$) and corresponding lengths (${L}_{1},{L}_{2},{L}_{3}$), mass ($m$) center of mass (${X}^{{CM}}$) and moment of inertia (${I}_{1}$) of the rotator, the diameter of the coil wire ($d$) and distance ($D$), angular span of each coil ($\Delta \theta$), the axial position of the top loop of the coil (${z}_{0}$), inner (${r}_{-}$) and outer (${r}_{+}$) radius, number of loops along the axial (${N}_{{{{\mathscr{L}}}}}$) and radial directions (${N}_{{{{\mathscr{R}}}}}$), and internal resistance (${R}^{{\mathbb{I}}}$).

**Fig. 4:**
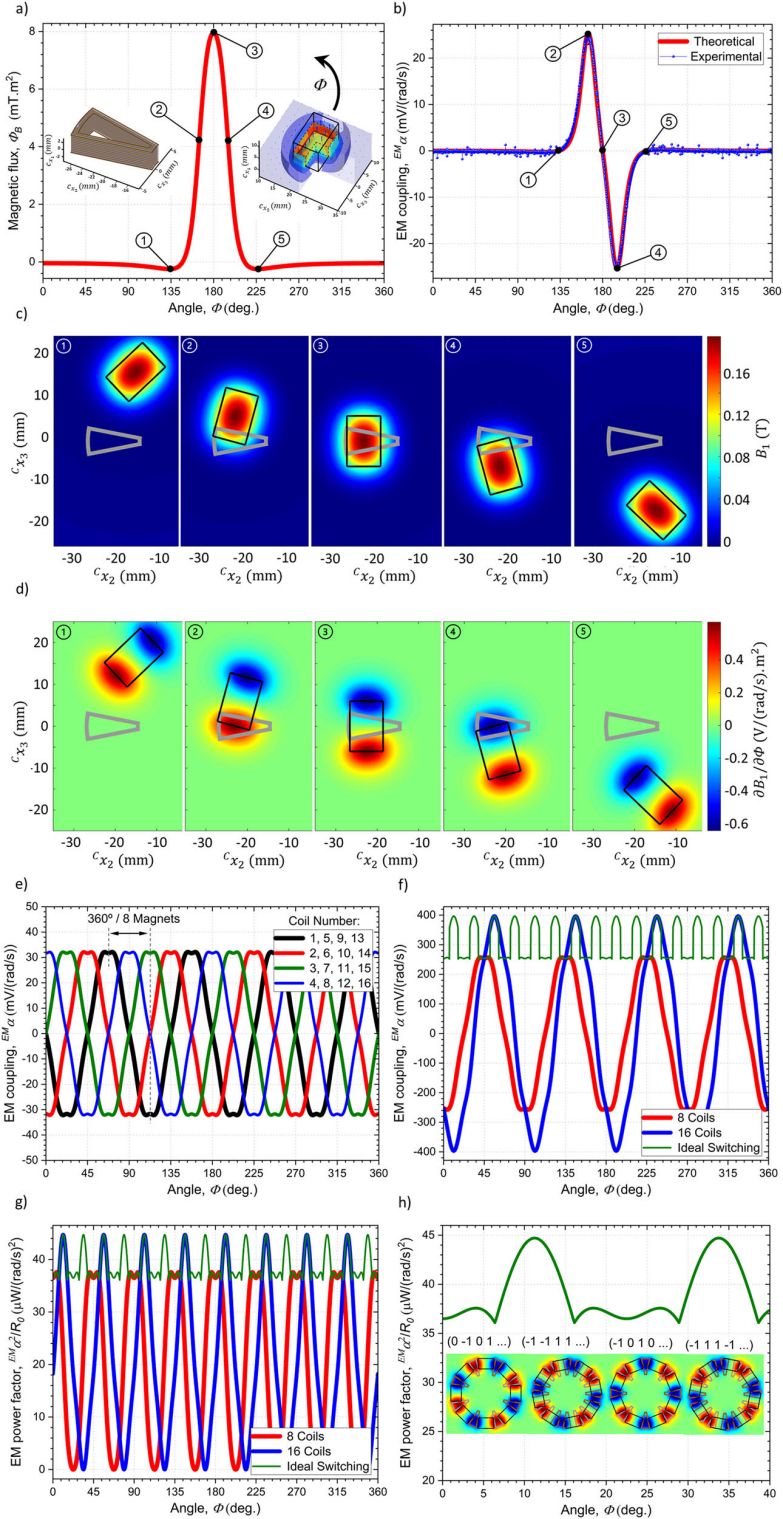
Characterization of the transduction mechanism of the adaptive harvester. **a** Magnetic flux produced by one of the harvester’s permanent magnets on one of its coils as a function of the $\varPhi$ angle between stator and rotator. The analytically described coil is represented in the inset. **b** Electromechanical coupling factor between one of the harvester’s permanent magnets and one of its coils. **c** Representation of the magnetic induction field in the axial direction in the transverse middle plane of the coils for several $\varPhi$ angles of interest. **d** Rate of change of the magnetic induction field in the axial direction with an infinitesimal change in the $\varPhi$ angle at different configurations. **e** Complete electromechanical coupling factor for each of the 16 coils and 8 magnets. **f** Total electromechanical coupling factor, and **g** power factor associated with: (i) one subdomain of 8 of the even-numbered coils permanently electrically connected in series between themselves; (ii) all the 16 coils connected in series; and (iii) dynamic rearrangement of the coils, dependent on the instantaneous position of the rotator in such a way as to always maximize the corresponding power conversion factor. **h** Detail of the electromechanical power factor associated with the coil switching scheme and corresponding optimal connection between coils.

In the case of the ${\mathbb{I}}$ th complete coil, with ${N}_{{{{\mathscr{L}}}}}$ loops along the axial direction and ${N}_{{{{\mathscr{R}}}}}$ loops along the radial direction, the electrical circuit equation can be obtained from the principle of superposition and Faraday’s law:

7 $${V}^{{\mathbb{I}}}=-{R}^{{\mathbb{I}}}{I}^{{\mathbb{I}}}-{L}_{{\mathbb{J}}}^{{\mathbb{I}}}{\dot{I}}^{{\mathbb{J}}}-{\!\,}^{{EM}}\alpha ^{{\mathbb{I}},1}\dot{\varPhi }{{;}}$$

$${\!\,}^{{EM}}\alpha ^{{\mathbb{I}},1}={\sum }_{{{{\mathscr{R}}}}=1}^{{N}_{{{{\mathscr{R}}}}}}{\sum }_{{{{\mathscr{L}}}}=1}^{{N}_{{{{\mathscr{L}}}}}}{\oint }_{{\partial S}^{{{{\mathscr{L}}}}{{{\mathscr{,}}}}{{{\mathscr{R}}}}}}\,\frac{\partial {\!\,}^{{M}}A_{I}}{\partial \varPhi }d{\!\,}^{C}X_{I},$$

where ${V}^{{\mathbb{I}}}$ is the output voltage, ${I}^{{\mathbb{I}}}$ the driving current, ${R}^{{\mathbb{I}}}$ the total internal resistance of the coil and ${L}_{{\mathbb{J}}}^{{\mathbb{I}}}$ the inductance matrix, ${\!\,}^{{EM}}\alpha ^{{\mathbb{I}},1}\left(\varPhi \right)$ is the EM coupling factor of the coil and the path integral is taken over the four segments described by Eq. (5) for all ${{{\mathscr{L}}}}$ and ${{{\mathscr{R}}}}$ indexed loops of the coil. This factor is strongly dependent on the $\varPhi$ angle as illustrated in Fig. 4a, b. The magnetic flux produced by a magnet on the coil in Fig. 4a shows that the flux is close to zero when the magnet is at its maximum distance from the coil and decreases slowly with an increasing angle $\varPhi$ up to a minimum at $\varPhi$ ~ 133.4°, where most of the magnetic field produced by the magnet is opposite to the axial direction. As the magnet gets closer to the coil, its most central and stronger field components pointing in the axial direction start to flow through the coil resulting in an increase in the total flux. A positive maximum of the flux is obtained when the magnet is exactly aligned with the coil at $\varPhi$ = 180°, with the flux subsequently dropping with the angle in accordance with the mirror symmetry of the geometry over the ${\!\,}^{C}x_{3}$ = 0 plane. Figure 4b shows the corresponding derivative of the flux curve, which according to Eq. (7) corresponds to the EM coupling factor. This factor attains a maximum value for an angle of $\varPhi$ ~ 164.8°, corresponding to the case where one of the faces of the magnet associated with a large change in induction field is approximately aligned with the centroid of the coil. A minimum value is reached when the opposite face is aligned with the centroid after the magnet moves by an angle approximately equal to its angular span relative to the center of the rotator. This coefficient was experimentally obtained by measuring the open-circuit output voltage from a single coil and magnet while rotating the EMG at a constant rate of 5 Hz (300 rpm). The results superimposed on the theoretically calculated ones in Fig. 4b exhibit a very good agreement.

The developed harvester comprised 8 magnets, with magnetizations sequentially pointing in different directions in such a way as to maximize the regions of large change of induction field previously depicted in Fig. 3d. Because of the 8^th^ fold symmetry of this configuration over the $\varPhi$ angle, from the superposition theorem the EM factor associated with the ${\mathbb{I}}$ th coil is a sum of the factors calculated using Eq. (7) and deviated by appropriate angles:

8 $${\!\,}^{{EM}}\alpha ^{{\mathbb{I}}}\left(\varPhi \right)={\sum }_{{\mathbb{K}}=1}^{8}{\left(-1\right)}^{{\mathbb{K}}-1}.{\!\,}^{{EM}}\alpha ^{{\mathbb{I}},1}\,\left({{{\mathrm{mod}}}}\left(\varPhi +[{\mathbb{K}}-1]\frac{2\pi }{8},2\pi \right)\right);$$

where ${{\mathrm{mod}}}$ is the modulo binary operation. The 16 coils of the harvester similarly have a 16^th^ fold symmetry over the $\varPhi$ angle, with the EM factor from each subsequent ${\mathbb{I}}$^th^ one being deviated by an angle of 360°/16. The calculated EM factor associated with each coil is shown in Fig. 4e, illustrating how the factors produced by a single magnet and coil in Fig. 4b are superimposed in the case of 8 magnets. Each coil can then be electrically connected in different ways between themselves and to an external circuit. If the coils are connected in series (described by an array ${\left(\pm 1\right)}_{{{{\mathscr{M}}}}}$, with $+1$, $-1,$ or $0$ as array elements and $+1$ representing the case with the $+$ and $\mbox{--}$ signs of an output circuit being connected to the $+$ and $\mbox{--}$ signs of the individual coil, $-1$ representing the opposite case, and $0$ representing a disconnected loop, as represented in Fig. 2a)) and to an external circuit, this yields a total voltage: $V={\sum }_{{\mathbb{I}}=1}^{\,16}{\left(\pm 1\right)}_{{\mathbb{I}}}{V}^{{\mathbb{I}}}$ and loop current: ${I}^{{\mathbb{I}}}={\left(\pm \!1\right)}_{{\mathbb{I}}}I$. Consequently, with Eqs. (7) and (8) the complete circuit equation of the EMG is:

9 $$V=-{R}_{0}I-{L}_{0}\dot{I}-{\!\,}^{{EM}}\alpha \, \dot{\varPhi }{{;}}$$

$${R}_{0}={\sum }_{{\mathbb{I}}=1}^{16}{R}^{{\mathbb{I}}};$$

$${L}_{0}={\sum }_{{\mathbb{J}}=1}^{16}{\sum }_{{\mathbb{I}}=1}^{16}{\left(\pm \!1\right)}_{{\mathbb{I}}}{L}_{{\mathbb{J}}}^{{\mathbb{I}}}{\left(\pm \!1\right)}_{{\mathbb{J}}};$$

$${\!\,}^{{EM}}\alpha \, \left(\varPhi \right)={\sum }_{{\mathbb{I}}=1}^{16}{\left(\pm \!1\right)}_{{\mathbb{I}}}{\!\,}^{{EM}}\alpha ^{{\mathbb{J}}=1}\left({{\mathrm{mod}}}\left(\varPhi +[{\mathbb{I}}\, {\mathbb{-}}\, 1]\frac{2\pi }{16},2\pi \right)\right),$$

where ${R}_{0}$ is the total internal resistance of the harvester, ${L}_{0}$ is the internal inductance and ${\!\,}^{{EM}}\alpha$ is the total EM coupling factor. A disconnected coil involves a corresponding open-circuit configuration with a null passing current, although if such coil is far away enough from the regions of maximum electromotive force generation in Fig. 3c it can also be approximated by a short-circuit configuration since the added Lorentz braking force will be negligible. Across a resistive load with $V={RI}$, and if the frequency of the output current in Eq. (9) is sufficiently small (i.e. $\omega \, \ll \, {R}_{0}/{L}_{0}$), this results in the circuit equation yielding a current proportional to the angular velocity of the rotator: $I\, \approx -\!\left[{\!\,}^{{EM}}\alpha /\left(R+{R}_{0}\right)\right]\dot{\varPhi }$. The instantaneous output power from the EMG thus takes approximately the form:

10 $$P={VI}=R{I}^{2}\, \approx \, \frac{R}{{\left(R+{R}_{0}\right)}^{2}}{\!\,}^{{EM}}a^{2}{\dot{\varPhi }}^{2},$$

exhibiting an increase with the square of the angular velocity of the rotator and the EM factor. The load resistance for a maximum output power and energy conversion efficiency is generally close to the internal resistance of the coil ${R}_{0}$, except under specific conditions of resonance where the $\dot{\varPhi }$ rate of rotation also tends to increase prominently with the total $R+{R}_{0}$ resistance, yielding a power conversion factor of: ${\!\,}^{{EM}}a^{2}/{R}_{0}$, which, in the case of this harvester with a single degree of freedom, completely quantifies the performance of the harvester independently of its rotation rate. The efficiency of energy conversion in a time spam between ${t}_{0}$ and ${t}_{1}$ can also be shown to take approximately the form^78^:

11 $$\eta \, \approx \, \frac {\int _{{t}_{0}}^{{t}_{1}}\frac{R}{{\left(R+{R}_{0}\right)}^{2}}{\!\,}^{{EM}}\alpha ^{2}{\dot{\varPhi }}^{2}{dt}}{\int_{{t}_{0}}^{{t}_{1}}\left[\frac{1}{\left(R+{R}_{0}\right)}{\!\,}^{{EM}}\alpha ^{2}+c\right]{\dot{\varPhi }}^{2}-{W}^{{Air}\to C}{dt}}{{{\rm{;}}}}$$

$${W}^{{Air}\to C}= -\!{c}^{{Air}-C}\left({\dot{T}}_{i}{\dot{T}}_{i}\right)-2{c}_{I}^{{Air}-C}\left({R}_{{Ki}}^{T}{\dot{T}}_{i}{\varepsilon }_{{KJI}}{\omega }_{J}\right) \\ -{c}_{{IJ}}^{{Air}-C}\left({\varepsilon }_{{KLI}}{\omega }_{L}{\varepsilon }_{{KMJ}}{\omega }_{M}\right),$$

where $c$ is a damping factor describing the mechanical friction between rotator and stator, which may depend on the angular velocity $\dot{\varPhi }$, and $-{W}^{{Air}\to C}$ the power loss due to friction between the stator and surrounding air with ${c}^{{Air}-C}$, ${c}_{I}^{{Air}-C}$ and ${c}_{{IJ}}^{{Air}-C}={c}_{{JI}}^{{Air}-C}$ its associated damping factors. Unlike in the case of the output power given by Eq. (10), if the effects of air friction are disregarded, the efficiency is shown to take a maximum value for a resistive load of: $R={R}_{0}\sqrt{1+\left({\!\,}^{{EM}}\alpha ^{2}/{R}_{0}c\right)}$, which increases with the power factor, and can potentially go up to 100% under such conditions in case the damping $c$ factor tends to 0. Overall, like the output power such conversion efficiency also increases with the aforementioned power conversion factor. According to Eq. (9), different configurations of the coils as described by the ${\left(\pm \!1\right)}_{{\mathbb{I}}}$ array are associated with different power factors over the $\varPhi$ angle of the system. Figure 4f and g depict the effective EM factor and power conversion factors for three relevant schemes of in-series connected coils, respectively. The first case (i) corresponds to having only one subdomain of even-numbered coils permanently electrically connected to an external circuit, i.e. with sequential coils connected in a $+1$ followed by a $-1$ scheme: ${\left(\pm \!1\right)}_{{\mathbb{I}}}=\left(010-1\ldots \right)$, which corresponds to always adding up the curves of Fig. 4e without ever having EM factors with opposite signs canceling each other. In the second case, (ii) the two subdomains of even and odd numbered coils are permanently active and connected in series between themselves with: ${\left(\pm \!1\right)}_{{\mathbb{I}}}=\left(11-1-1\ldots \right)$. Finally, the third case (iii) incorporates a dynamic rearrangement of the coils dependent on the instantaneous position of the rotator in such a way as to always maximize the corresponding power conversion factor, i.e. with all allowed transitions $1\leftrightarrow 0\leftrightarrow -1$ in the ${\left(\pm \!1\right)}_{{\mathbb{I}}}$ array. As shown in Fig. 4d, case (ii) is associated with a larger peak-to-peak amplitude of the EM factor compared to (i), although also having a two times larger total internal resistance ${R}_{0}$. The dynamic coil switching (iii), as opposed to the schemes (i) and (ii), yields a relatively large and almost constant factor over all the $\varPhi$ positions, while the number of active coils alternately switches between 8 and 16, corresponding to each subdomain or both, followed by an equivalent switch in internal resistance. Figure 4g shows that the power factors respectively attain a maximum value of ~38 μW (rad/s)^-2^ and ~45 μW (rad/s)^-2^ and sequentially drop down to zero in the (i) and (ii) configurations. These two configurations have an equivalent average of the power factor over the $\varPhi$ angle of the order of ~20 μW (rad/s)^-2^. The dynamic coil switching scheme in the ideal case is shown to permit increasing this average factor by up to two-fold. Accordingly, for the same rotation rate this coil switching approach should result in an up to $x=2$ times increase in the output power and an energy conversion efficiency under optimal load conditions multiplied by a factor of $\eta /{\eta }_{0}$ ~ $2x/[g+({\eta }_{0}-1)\sqrt{g}-2{\eta }_{0}x]$, $g=2{\eta }_{0}\left(2x-1\right)+{\eta }_{0}^{2}+1$, with ${\eta }_{0}$ being the efficiency (between 0 and 1) obtained without employing the coil switching mechanism. Unlike in the static cases, with the coil switching the power conversion factor will be relatively large over all the configurations of the system, thus always adapting the EMG to provide maximum energy conversion efficiency. Furthermore, the control circuitry can also be engineered to provide automatic rectification of the voltage output if the direction of rotation or the sign of the pickup open-circuit voltage is determined. Figure 4h details the optimum configurations of the coils for each position of the rotator, from which it is possible to see that only coils that pick up a sufficiently large change in magnetic flux are activated at each angle, such that it maximizes the power being sent to the external circuit and minimize the Ohmic losses in the internal resistance of the device (see Supplementary Movie 3).

### Results using simple rotations

**Fig. 5:**
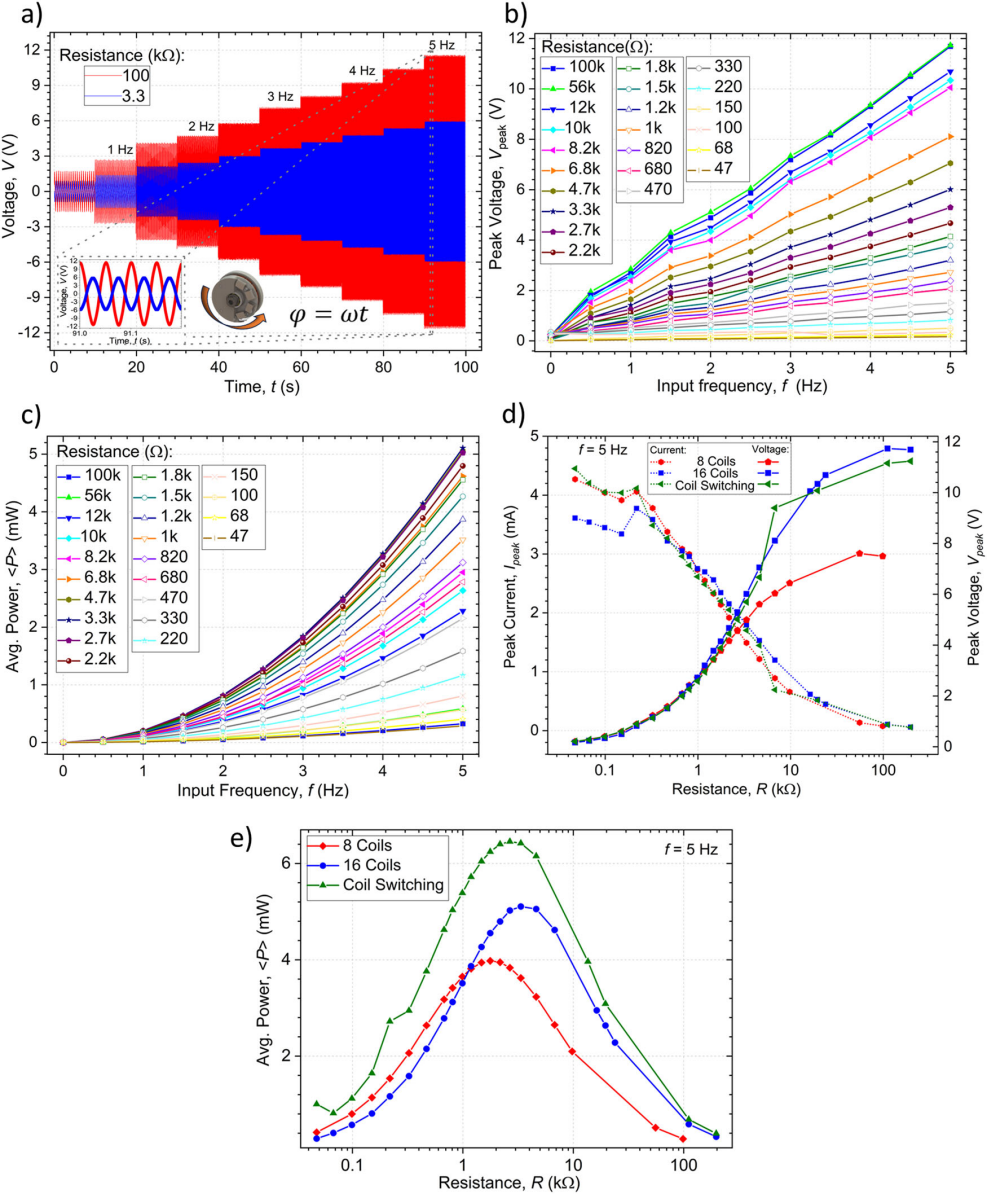
Electric characterization related to energy harvesting using simple rotations. **a** Experimental measurement of the output voltage variation of the harvester over time for a continuous rotor rotation input, applying an increasing frequency and for a load resistance of 100 kΩ (maximum measured resistance) and 3.3 kΩ (optimum resistance for these test conditions), with all the 16 coils of the system connected in series. **b** Variation of the peak voltage as a function of frequency in the system with all 16 coils connected. **c** Average output power in the system with all coils connected. **d** Experimental peak voltage (right scale) and experimental peak current (left scale) as a function of the resistance implemented in the system (comparison between experimental tests and theoretical results). **e** Average output power as a function of the load resistance for the different types of electrical configurations and theoretical results.

Experimental measurements of the output voltages over time were conducted in response to an input rotation with increasing frequency. Figure 5a illustrates the voltage measured as a function of time for the system with 16 permanently active coils and two loads, with 100 kΩ corresponding to the maximum resistance measured and 3.3 kΩ corresponding to the resistance for which the average power is maximum. As shown, the output signal, by virtue of Faraday’s law of electromagnetic induction, has a frequency proportional to that of the input signal. The frequency of the output is multiplied four-fold in the case of the 8 and 16 active coils according to the 90° rotation symmetry of the harvester, as is better illustrated in the inset of Fig. 4h. The peak values of the output voltage and average power were analyzed relative to the frequency of the input rotation, with different load resistances. The results obtained are recorded in the graphical representations of Figs. 5b and c, for the case of 16 permanently active coils, respectively showing a linear and cubic increase with the angular velocity in accordance with Eq. (9) and Eq. (10). Figure 5d and e summarize the results of the experiments by depicting, respectively, the peak voltage/current and average powers measured for a rotation rate of 5 Hz and as a function of the resistive loads and comparison of the experimental values using the two coil configurations with the theoretical results in the case of the system operating with the switching configuration. In agreement with Eq. (10), under such simple rotation, the RH is shown to behave like a linear open-circuit voltage source in series with an internal ${R}_{0}$ load. The peak voltage for all tested coil configurations increases with increasing resistance while the peak current decreases. The maximum output powers were also obtained for loads close to the internal resistance of the circuits in each of the tested configurations. Maximum open-circuit peak voltages of ${V}_{{OC}}$ ~11.4 V were obtained for the systems with 16 coils, as modulated by the EM factors shown in Fig. 4f. The configuration with 16 permanently active coils had twice the internal resistance of that with the 8 coils, and thus the peak short-circuit currents took approximately the form of: ${I}_{{SC}}={V}_{{OC}}/{R}_{0}$, having values of 3.6 mA and 4.3 mA, respectively. Overall, the configuration with 16 coils yielded under optimal load conditions average output powers of up to 5.1 mW, while this was just of 4 mW for the system with 8 coils.

### Results using general 3D mechanical excitations

If the stator is subjected to mechanical forces and torques from the environment and the rotator and eccentric mass are free to move, applying the balance laws of linear and angular momentum to the rigid stator and rotator described by Eq. (1) results in the set of 12 ordinary differential equations for general movements in 3D space^78^:

12 $$m\left[{R}_{{Ii}}^{T}\ddot{{T}_{i}}+{\varepsilon }_{{IJK}}{\dot{\omega }}_{J}{\delta }_{K}+{\varepsilon }_{{IJK}}{\omega }_{J}{\varepsilon }_{{KLN}}{\omega }_{L}{\delta }_{N}\right.\\ \left.+\, {R}_{\varPhi I{I}^{{\prime} }}({\varepsilon }_{{I}^{{\prime} }{J}^{{\prime} }{K}^{{\prime} }}{\dot{\omega }}_{{J}^{{\prime} }}^{\varphi \varPhi }{\!\,}^{M}X_{{K}^{{\prime} }}^{{CM}} +{\varepsilon }_{{I}^{{\prime} }{J}^{{\prime} }{K}^{{\prime} }}{\omega }_{{J}^{{\prime} }}^{\varphi \varPhi }{\varepsilon }_{{K}^{{\prime} }{L}^{{\prime} }{N}^{{\prime} }}{\omega }_{{L}^{{\prime} }}^{\varphi \varPhi }{\!\,}^{M}X_{{N}^{{\prime} }}^{{CM}})\right]\\ ={F}_{I}^{{EM},C\to M}+{F}_{I}^{{Const},C\to M}+{F}_{I}^{{Fric},C\to M}+{F}_{I}^{{Air}\to M}+{F}_{I}^{{Grav},M};$$

$${\!\,}^{C}m\left[{R}_{{Ii}}^{T}\ddot{{T}_{i}}+{\varepsilon }_{{IJK}}{\dot{\omega }}_{J}{\!\,}^{C}X_{K}^{{CM}}+{\varepsilon }_{{IJK}}{\omega }_{J}{\varepsilon }_{{KLN}}{\omega }_{L}{\!\,}^{C}X_{N}^{{CM}}\right]\\ =-{F}_{I}^{{EM},C\to M}-{F}_{I}^{{Const},C\to M}-{F}_{I}^{{Fric},C\to M}+{F}_{I}^{{Air}\to C}+{F}_{I}^{{Grav},C}+{F}_{I}^{{Ext},C};$$

$$\;{R}_{\varPhi I{I}^{{\prime} }}\left[{\varepsilon }_{{I}^{{\prime} }{J}^{{\prime} }{K}^{{\prime} }}{\omega }_{{J}^{{\prime} }}^{\varphi \varPhi }{I}_{{K}^{{\prime} }{L}^{{\prime} }}^{{CM}}{\omega }_{{L}^{{\prime} }}^{\varphi \varPhi }+{I}_{{I}^{{\prime} }{J}^{{\prime} }}^{{CM}}{\dot{\omega }}_{{J}^{{\prime} }}^{\varphi \varPhi }\right]\\ ={\tau }_{I}^{{EM},C\to M}+{\tau }_{I}^{{Const},C\to M}+{\tau }_{I}^{{Fric},C\to M}+{\tau }_{I}^{{Air}\to M}+{\tau }_{I}^{{Grav},M};$$

$${\varepsilon }_{{IJK}}{\omega }_{J}{{\!\,}^{C}I}_{{KL}}^{{CM}}{\omega }_{L}+{{\!\,}^{C}I}_{{IJ}}^{{CM}}{\dot{\omega }}_{J}=-{\tau }_{I}^{{EM},C\to M}-{\tau }_{I}^{{Const},C\to M}-{\tau }_{I}^{{Fric},C\to M}\\ -{\varepsilon }_{{IJK}}\left({\delta }_{J}+{R}_{\varPhi J{J}^{{\prime} }}{\!\,}^{M}X_{{J}^{{\prime} }}^{{CM}}-{\!\,}^{C}X_{J}^{{CM}}\right)\left({F}_{K}^{{EM},C\to M}+{F}_{K}^{{Const},C\to M}+{F}_{K}^{{Fric},C\to M}\right)\\ +{\tau }_{I}^{{Air}\to C}+{\tau }_{I}^{{Grav},C}+{\tau }_{I}^{{Ext}\to C},$$

where $m$ and ${\!\,}^{C}m$ are the masses of the rotator and stator, respectively, ${I}_{{I}^{{\prime} }{J}^{{\prime} }}^{{CM}}$ and ${{\!\,}^{C}I}_{{IJ}}^{{CM}}$ are the moment of inertia matrices of the rotator and stator in relation to its respective centers of mass, ${\!\,}^{M}X_{{I}^{{\prime} }}^{{CM}}$ and ${\!\,}^{C}X_{I}^{{CM}}$ are the center of mass positions of the rotator and stator, respectively, ${\omega }_{I}$ are the components of the angular rotation vector of the stator and ${\omega }_{{I}^{{\prime} }}^{\varphi \varPhi }={\omega }_{{I}^{{\prime} }}^{\varPhi }+{R}_{\varPhi {I}^{{\prime} }I}^{T}{\omega }_{I}$ is the angular velocity of the rotator in relation to the inertial frame (${\dot{\omega }}_{{I}^{{\prime} }}^{\varphi \varPhi }={\dot{\omega }}_{{I}^{{\prime} }}^{\varPhi }+{R}_{\varPhi {I}^{{\prime} }I}^{T}{\dot{\omega }}_{I}+{{\varepsilon }_{{I}^{{\prime} }{J}^{{\prime} }{K}^{{\prime} }}R}_{\varPhi {J}^{{\prime} }J}^{T}{\omega }_{J}{\omega }_{{K}^{{\prime} }}^{\varPhi }$). ${F}_{I}^{{EM},C\to M}$ is the electromechanical force exerted by the stator on the rotator (i.e. coils on the magnets), ${F}_{I}^{{Const},C\to M}$ and ${F}_{I}^{{Fric},C\to M}$ are respectively the constrain and friction forces between the stator and rotator, responsible for limiting the number of degrees of freedom of the system, ${F}_{I}^{{Air}\to M}$ and ${F}_{I}^{{Air}\to C}$ are friction forces produced by the air on the rotator and stator, ${F}_{I}^{{Grav},M}$ and ${F}_{I}^{{Grav},C}$ are the gravitational forces applied to the rotator and stator and ${F}_{I}^{{Ext},C}$ is and external mechanical contact force applied to the stator. The ${\tau }_{I}$ components represent the torques relative to the respective centers of mass associated with the same previous forces. The friction tractions, responsible for the friction forces and torques, can be considered to be proportional to the difference between the velocities across the interfaces of contact, and the constraint tractions to span the vector space that is not already spanned by all the allowed velocity difference vectors. Accordingly, it can be shown that the constraint torque in the axial direction produced by tractions in the interface between the stator and rotator depends only on the constraint forces: ${\tau }_{I=1}^{{Const},C\to M}=-{\varepsilon }_{1{JK}}{R}_{\varPhi J{J}^{{\prime} }}{\!\,}^{M}X_{{J}^{{\prime} }}^{{CM}}{F}_{K}^{{Const},C\to M}$. The third balance of the angular momentum equation, counting from the top, for the rotator in Eq. (12) can thus be combined with the first balance of the linear momentum equation for the rotator in order to remove the constraint components. In accordance with the geometry of the designed harvester depicted in Fig. 1a,b and Fig. 2a, b we have: ${\delta }_{I}={\delta }_{I1}\delta$, with a time-independent $\delta$, and ${\!\,}^{C}X_{I}^{{CM}}=0$. Due to reflection symmetries in the ${\!\,}^{M}x_{1}=0$ and ${\!\,}^{M}x_{2}=0$ planes of the rotator, the co-moving body basis ${\!\,}^{M}\hat{{{{\boldsymbol{x}}}}}_{{I}^{{\prime} }}$ vectors must correspond to the principal axis of the rotator such that the center of mass must lie along the ${\!\,}^{M}\hat{{{{\boldsymbol{x}}}}}_{3}$ direction: ${\scriptstyle{M}\atop }\!X_{I'}^{CM}=-{\delta }_{I'3}{X}^{CM}$, and the moment of inertia matrix relative to the origin of this frame is diagonal with elements: ${I}_{11}={I}_{1}$, ${I}_{22}={I}_{2}$ and ${I}_{33}={I}_{3}$, and associated with the inertia matrix in Eq. (12) through: ${I}_{{I'}{J'}}^{{CM}}={I}_{{I'}{J'}}-m({\scriptstyle{M}\atop }\!X_{{K'}}^{{CM\,M}}X_{{K'}}^{{CM}}{\delta }_{{I'}{J'}}{-}^{M}X_{{I'}}^{{CM\,M}}X_{{J'}}^{{CM}})$. Since the rotator is approximately semi-cylindrical we note that: ${I}_{2}\, \approx \, {I}_{3}$. The force and torque components can be given by: ${\tau }_{I}^{{EM},C\to M}+{\varepsilon }_{{IJK}}{R_{\varPhi J{J'}}}^{M}X_{{J}^{{\prime} }}^{{CM}}{F}_{K}^{{EM},C\to M}={R_{\varPhi II'}}^{{EM}}\alpha _{{I'}}I$, $({\tau }_{1}^{{Fric},C\to M}+{\tau }_{1}^{{Air},C\to M})+{\varepsilon }_{1{JK}}{R_{\varPhi J{J'}}}^{M}X_{{J'}}^{{CM}}({F}_{K}^{{Fric},C\to M}+{F}_{K}^{{Air},C\to M})=-c \dot {\varPhi } ,$ ${F}_{I}^{{Grav},M}=-{mg}{R}_{3I}$ and ${\tau }_{I}^{{Grav},M}=0$ with $g$ the standard acceleration of gravity and $c$ a damping factor. With these considerations, the general equation of motion of the pendulum harvester with prescribed time-dependent 3D translations and rotations of the stator becomes:

13 $${I}_{1}\ddot{\varPhi }\, = \, {\tau }_{0}+{\tau }_{1}\sin \left(\varPhi \right)+{\tau }_{2}\cos \left(\varPhi \right)+{\tau }_{3}\sin \left(2\varPhi \right)+{\tau }_{4}\cos \left(2\varPhi \right)\\ -c\dot{\varPhi }+{\!\,}^{{EM}}\alpha \, I{{{\rm{;}}}}$$

$${\tau }_{0}\left(t\right)=-{I}_{1}{\dot{\omega }}_{1};$$

$${\tau }_{1}\left(t\right)=-m{X}^{{CM}}\left[{R}_{3i}^{T}\left({\ddot{T}}_{i}+{\delta }_{3i}g\right)+\delta \left(-{\dot{\omega }}_{2}+{\omega }_{1}{\omega }_{3}\right)\right];$$

$${\tau }_{2}\left(t\right)=-m{X}^{{CM}}\left[{R}_{2i}^{T}\left({\ddot{T}}_{i}+{\delta }_{3i}g\right)+\delta \left({\dot{\omega }}_{3}+{\omega }_{1}{\omega }_{2}\right)\right];$$

$${\tau }_{3}(t)=\left({I}_{2}-{I}_{3}\right)\frac{1}{2}\left({\omega }_{3}^{2}-{\omega }_{2}^{2}\right);$$

$${\tau }_{4}\left(t\right)=\left({I}_{2}-{I}_{3}\right){\omega }_{2}{\omega }_{3},$$

with ${I}_{1}={I}_{11}^{{CM}}+m{{X}^{{CM}}}^{2},$ and which can be solved together with the circuit Eq. (9) and given the relation between the voltage and current in the external circuit as well as initial conditions for the current $I\left(0\right)$, angle $\varPhi (0)$ and angular velocity $\dot{\varPhi }(0)$. The term ${R}_{{Ii}}^{T}\left({\ddot{T}}_{i}+{\delta }_{3i}g\right)$ represents the proper acceleration of the harvester in the ${\!\,}^{C}\hat{{{{\boldsymbol{x}}}}}_{I}$ direction. The inertial force in Eq. (13), partially responsible for setting the rotator into motion relative to the stator, is shown to have translational components in the form of ${R}_{{Ii}}^{T}{\ddot{T}}_{i}$ together with centrifugal forces proportional to square powers of the angular velocities and Euler forces proportional to time derivatives of such angular velocities. The form of Eq. (13) indicates that the rotator may be set into motion, and thus generate power, in response to time-changing translations along the non-axial ${\!\,}^{C}\hat{{{{\boldsymbol{x}}}}}_{2}$ and ${\!\,}^{C}\hat{{{{\boldsymbol{x}}}}}_{3}$ directions and mostly due to rotations around the axial direction ${\!\,}^{C}\hat{{{{\boldsymbol{x}}}}}_{1}$ which result in time-changing torques produced by the gravitational field. In the low-frequency approximation and with a resistive load the ${\!\,}^{{EM}}\alpha I$ term simplifies to a damping torque $-{c}_{{EM}}\dot{\varPhi }$ with factor ${c}_{{EM}}={\!\,}^{{EM}}{\alpha \, (\varPhi )}^{2}/\left(R+{R}_{0}\right)$, which decreases with an increasing load. The presence of this term indicates that the peak angular velocities and thus the gains from the coil switching approach in the pendulum regime are not expected to be as high as the ones discussed in the previous section. The total damping factor is thus: ${c}_{\varPhi }=c+{c}_{{EM}}$. Linearizing the sine and cosine terms of the $\varPhi$ angle results in a motion equation similar to that of a forced harmonic parametric oscillator with a resonant behavior and a time-dependent natural frequency. The non-linear restoring force is proportional to: $k=-\partial {I}_{1}\ddot{\varPhi }/\partial \varPhi =-{\tau }_{1}\cos \left(\varPhi \right)+\, {\tau }_{2}\sin \left(\varPhi \right)-2{\tau }_{3}\cos \left(2\varPhi \right)+2{\tau }_{4}\sin \left(2\varPhi \right)$, with $k$ being the equivalent elastic stiffness constant and ${\omega }_{0}=\sqrt{k/{I}_{1}}$ the natural angular frequency.

**Fig. 6:**
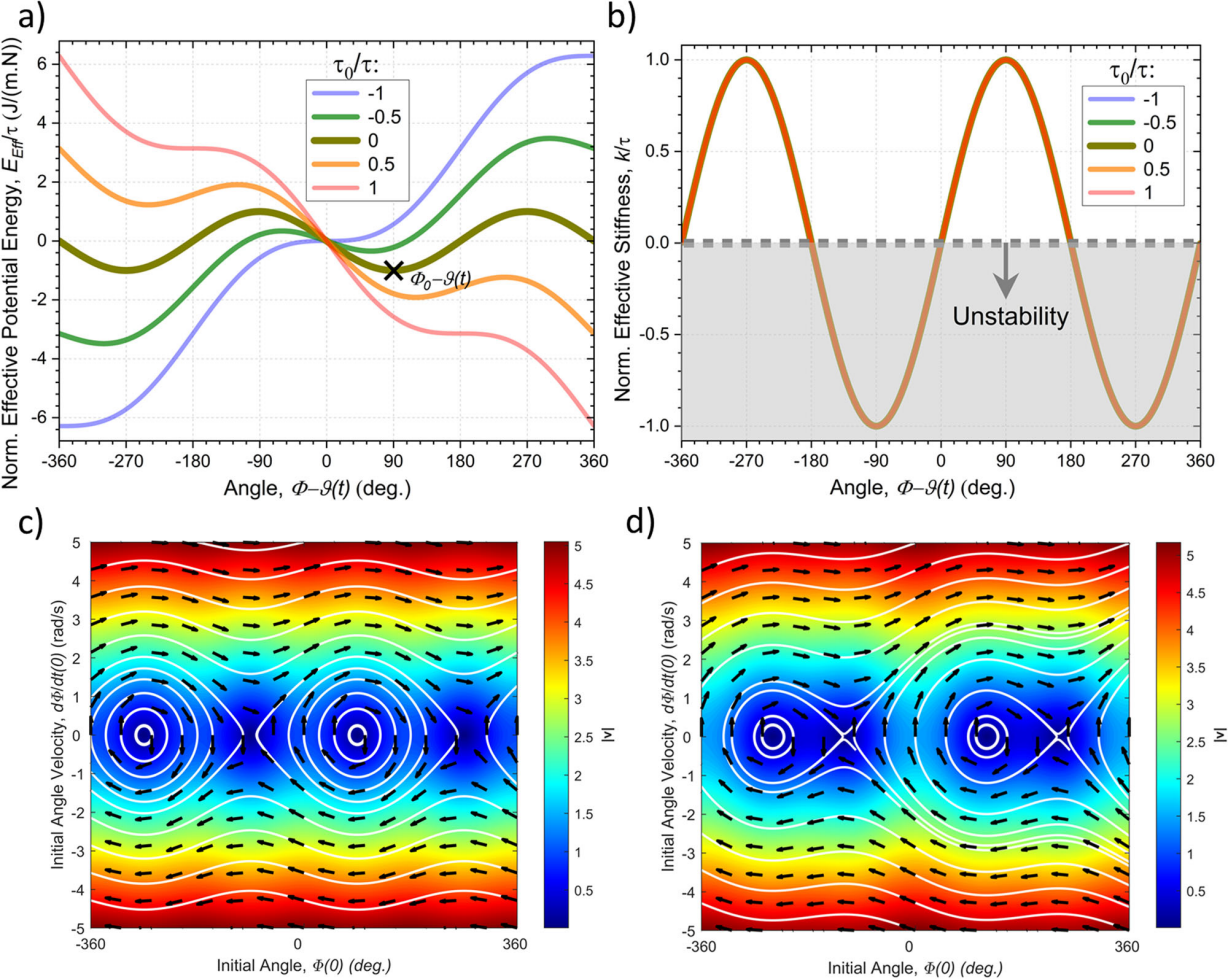
Dynamics of the eccentric mass of the harvester under the effects of constant applied 3D general torques (as described by Eq. (13)). **a** Equivalent potential energy, and **b** elastic stiffness of the system as a function of the angular configuration for various values of ${\tau }_{0}/\tau$. Phase portrait of the rotator with arrows indicating the direction ${{{\bf{v}}}}\, {{{\boldsymbol{=}}}}\, (\dot{\varPhi },\ddot{\varPhi })$ of the time-evolution of the system for a given initial angle and angular velocity with torques: $\tau /{I}_{1}$ = 1, $\vartheta$ = 0, and **c**. ${\tau }_{0}/\tau$ = 0, and **d**. ${\tau }_{0}/\tau$ = 0.5. The colour scale shows the amplitude of the variation. The white lines represent some of the characteristic phase paths without damping.

In the case of constant ${\tau }_{a}$ torque factors in Eq. (13), the system has a static equilibrium for an angle $\varPhi ={\varPhi }_{0}$ satisfying: ${\tau }_{0}+{\tau }_{1}\sin \left({\varPhi }_{0}\right)+{\tau }_{2}\cos \left({\varPhi }_{0}\right)+{\tau }_{3}\sin \left(2{\varPhi }_{0}\right)+{\tau }_{4}\cos \left(2{\varPhi }_{0}\right)=0$, which are the roots of a degree 4 polynomial equation. In the simpler case considering: ${\tau }_{3}\, \approx \, {\tau }_{4}\, \approx \, 0$, the system has at most two critical points (considering ${\tau }_{2}\, \ne \, 0$): ${\varPhi }_{0}=\vartheta \pm {\cos }^{-1}\left(-{\tau }_{0}/\tau \right)$, with ${\tau }^{2}={\tau }_{1}^{2}+{\tau }_{2}^{2}$ and $\vartheta ={{{\rm{atan}}}}2\left({\tau }_{1},{\tau }_{2}\right)$. Two solutions exist for: $|{\tau }_{0}| < \tau$, and no solutions otherwise with the system being in constant motion. Stability analysis of Eq. (13), performed by inputting the static solution ${\varPhi }_{0}$ together with an added small perturbation $\Delta \varPhi \left(t\right)$ term and linearizing the torques results in a differential equation with the solution: $\Delta \varPhi \left(t\right)={C}_{1}{e}^{i{\omega }_{0}t}+{C}_{2}{e}^{-i{\omega }_{0}t}$, with ${\omega }_{0}=\sqrt{\tau \sin \left({\varPhi }_{0}-\vartheta \right)/{I}_{1}}$, which has terms that increase exponentially with time for an imaginary ${\omega }_{0}$. Together with the static equilibrium condition this shows that the steady state for which: $0 \, < \, {\varPhi }_{0}-\vartheta \, < \, \pi$ (i.e. ${\varPhi }_{0}=\vartheta +{\cos }^{-1}\left(-{\tau }_{0}/\tau \right)$), is stable, while the one for which: $-\pi \, < \, {\varPhi }_{0}-\vartheta \, < \, 0$ (i.e. ${\varPhi }_{0}=\vartheta -{\cos }^{-1}\left(-{\tau }_{0}/\tau \right)$), is unstable as shown in Fig. 6b. Accordingly, the harvester should always have at most a single stable equilibrium state to which it is attracted. The stiffness for such a point increases with the $\varPhi$ angle up to $\vartheta +\pi /2$ and subsequently decreases between $\vartheta +\pi /2$ and $\vartheta +\pi$. The natural angular frequency thus follows a concave curve varying between $0$ and $\sqrt{\tau /{I}_{1}}$, and the system in general has a softening stiffness. Therefore, it behaves similarly to a Duffing oscillator having a hysteretic transfer function with a resonant state overhanging to the lower frequencies when down-sweeping the frequency. The effective potential energy of the harvester can be given by: ${U}_{{Eff}}=-\int {\tau }_{0}+{\tau }_{1}\sin \left(\varPhi \right)+{\tau }_{2}\cos \left(\varPhi \right)\, d\varPhi =-\tau [\left({\tau }_{0}/\tau \right)\left(\varPhi -\vartheta \right)+\sin \left(\varPhi -\vartheta \right)]$, which, evidently, has a minimum at ${\varPhi }_{0}-\vartheta =\pi /2$ for a null ${\tau }_{0}/\tau$ ratio and moves to higher or lower values as ${\tau }_{0}/\tau$ increases or decreases, respectively, as depicted in Fig. 6a. For a given relatively small constant total energy of the rotator, without damping losses, it will tend to oscillate back a fourth around the minimum of potential energy taking a maximum in kinetic energy at such minimum and a null value at the intersection with the potential curve. Figure 6c and d show the phase portrait of the EMG when subjected to a general translation and/or rotation with time-constant ${\tau }_{0}$, ${\tau }_{1}$ and ${\tau }_{2}$ terms in Eq. (13) and $\tau /{I}_{1}$ = 1, $\vartheta$ = 0, ${\tau }_{0}/\tau$ = 0 and ${\tau }_{0}/\tau$ = 0.5. This evidences the presence of a stable attractor for an angle of ${\varPhi }_{0}={\cos }^{-1}\left(-{\tau }_{0}/\tau \right)$, around which the rotator will tend to oscillate following the indicated phase paths in case there is no damping. With non-null damping, the system should tend to spiral towards the attractor. The figures also show that, as the initial velocity at the stable angle, increases, the rotator can progressively attain increasing maximum angles until it starts to rotate continuously above a sufficiently large velocity. For non-null ${\tau }_{0}/\tau$, the absolute value of the velocity of the rotator can also increase progressively (up to infinity in this non-relativistic approximation). Multiplying Eq. (13) with the angular velocity $\dot{\varPhi }$ and combining with the circuit Eq. (9) results in a statement of conservation of energy:

14 $$\frac{d}{{dt}}\left(K+{U}_{{Eff}}+\frac{1}{2}{L}_{0}{I}^{2}\right)=-c{\dot{\varPhi }}^{2}-{VI}-{R}_{0}{I}^{2},$$

where $K=(1/2){I}_{1}{\dot{\varPhi }}^{2}$ is the kinetic energy of the rotator as seen from the frame of the stator and $(1/2){L}_{0}{I}^{2}$ is the energy contained in the inductance, while ${VI}$ is the power sent to the external circuit, ${R}_{0}{I}^{2}$ the power loss due to Joule heating in the internal resistance of the coil, and $c{\dot{\varPhi }}^{2}$ the power loss due to friction. The time derivative of the effective potential energy should thus contain the input mechanical power from the environment. Since the difference of potential energy between the minimum and maximum peak values decreases with the $|{\tau }_{0}/\tau |$ ratio as: $\Delta {U}_{{Eff}}=2\tau \left[\sqrt{1-{({\tau }_{0}/\tau )}^{2}}-|{\tau }_{0}/\tau |{\cos }^{-1}\left(|{\tau }_{0}/\tau |\right)\right]$, the pendulum in this case can more easily cross the unstable maximum peak state potentially resulting in continuous rotations or chaotic dynamics. Consequently, the minimum angular velocity of the rotator to be able to overcome the potential barrier from the equilibrium state should be: $\dot{\varPhi }=2\sqrt{\tau /{I}_{1}}\sqrt{(\Delta {U}_{{Eff}}/2\tau )}$. In the case of relatively small applied accelerations, the natural frequency is constant ${\omega }_{0}\, \approx \, \sqrt{m{X}^{{CM}}g/{I}_{1}}\, \approx \, 2\pi .1.55\,{{{\rm{rad}}}}.{{{\rm{Hz}}}}$ and the EMG behaves like a simple gravitational pendulum. For small angular displacement relative to the equilibrium state $\varPhi -{\varPhi }_{0}$, the system also satisfies the linear forced oscillator equation: $\ddot{\varPhi }+2{\omega }_{0}\zeta \dot{\varPhi }+{\omega }_{0}^{2}\varPhi ={\omega }_{0}^{2}{\varPhi }_{0}(t)$, with $\zeta ={c}_{\varPhi }/2{\omega }_{0}{I}_{1}$ and ${c}_{\varPhi }=c+{\!\,}^{{EM}}{\alpha ({\varPhi }_{0})}^{2}/\left(R+{R}_{0}\right)$, which has a known analytical solution.

As an example, considering the case of a simple rotation with a constant rate of the stator by an angle $\varphi =\omega t$ around the axial direction (with a rotation matrix ${{{\boldsymbol{R}}}}(t)$ equivalent to that in Eq. (2) with the transformation $\varPhi \to \varphi$) and a pendulum-like translation of: ${T}_{i}={l}_{0}(0,\sin \left(\omega t\right),-\cos \left(\omega t\right))$, with ${l}_{0}$ the constant distance from the rotation pivot point to the center of the stator, as depicted in Fig. 2c, Eq. (13) results in: ${\tau }_{0}=0$, ${\tau }_{1}=-m{X}^{{CM}}\left({l}_{0}{\omega }^{2}+g\cos \left(\omega t\right)\right)$ and ${\tau }_{2}=-m{X}^{{CM}}\left(g\sin \left(\omega t\right)\right)$. Consequently ${\varPhi }_{0}=\vartheta \pm \pi /2$ and at low frequencies ($\omega \, \ll \, {\omega }_{0}$) the gravity torque dominates and the stable angle changes linearly with time: ${\varPhi }_{0}=-\omega t$, so that the eccentric mass tends to be pulled towards the lowest point relative to the ground (i.e. $\varPhi +\varphi =0$). The unstable state, on the other hand, occurs at the highest point: ${\varPhi }_{0}=-\omega t+\pi$. Taking the effects of damping into account, in the low angular displacement approximation a steady state solution is: $\varPhi =-\omega t+2\zeta (\omega /{\omega }_{0})$, which indicates that the angle follows the minimum of effective potential energy with a phase lag of $2\zeta (\omega /{\omega }_{0})$ that increases with the frequency and is related to the power loss due to damping. Furthermore, at higher frequencies ($\omega \, \gg \, {\omega }_{0}$) the centrifugal force represented by the term ${l}_{0}{\omega }^{2}$ starts to dominate and the stable state tends toward zero ${\varPhi }_{0}\to 0$, so that the eccentric mass is always pulled towards the furthest point from the pivot of rotation. Because this minimum of effective potential energy doesn’t change with time, the $\varPhi$ angle remains static and thus the harvester produces no electric power. The centrifugal torques, more significant for large rotation ratios, are in general a liability to the performance of the EMGs since they hamper the relative rotation between rotator and stator and thus the conversion of electrical power. This kind of pendular architecture is therefore best targeted at relatively low-frequency applications.

#### Harmonic translation excitation

A mechanical harmonic translation applied to the stator in the horizontal ${\!\,}^{C}\hat{{{{\boldsymbol{x}}}}}_{2}$ direction with: ${T}_{i}={\delta }_{i2}X\cos \left(\omega t\right)$ and ${{{\boldsymbol{R}}}}={{{\boldsymbol{I}}}}$, ${{{\boldsymbol{I}}}}$ being the identity matrix, was tested with $X$ = 20 mm and by sweeping the input frequency. With this input movement, the equation of motion in Eq. (13) simplifies to:

15 $${I}_{1}\ddot{\varPhi }=-m{X}^{{CM}}\left[g\sin \left(\varPhi \right)-{\omega }^{2}X\cos \left(\omega t\right)\cos \left(\varPhi \right)\right]-c\dot{\varPhi }+{\!\,}^{{EM}}\alpha I.$$

The angle of stable equilibrium of the system is given by: ${\varPhi }_{0}=\vartheta +\pi /2\,={\tan }^{-1}\left({\omega }^{2}X\cos \left(\omega t\right)/g\right)$, thus oscillating between up to $-\pi /2$ and $\pi /2$ at the larger frequencies. In the low angle of displacement approximation, with a complex load $Z$, this equation has an analytic solution represented by the steady-state phasor (using complex algebra: $i=\sqrt{-1}$; $\hat{z}={z}^{{\prime} }+i{z}^{{\prime} {\prime} }$; ${z}^{{\prime} },{z}^{{\prime} {\prime} }{\mathbb{\in }}\, {\mathbb{R}}$; ${\hat{z}}^{* }={z}^{{\prime} }-i{z}^{{\prime} {\prime} }$; ${\left|\hat{z}\right|}^{2}=\hat{z}{\hat{z}}^{* }={z}^{{\prime} 2}+{z}^{{\prime} {\prime} 2}$; $\hat{z}=\left|\hat{z}\right|{e}^{i\vartheta }$ ; $z=(1/2)(\hat{z}{e}^{i\omega t}+{\hat{z}}^{* }{e}^{-i\omega t})={z}^{{\prime} }\cos (\omega t)-{z}^{{\prime} {\prime} }\sin (\omega t)=\,\left|\hat{z}\right|\cos (\omega t+\vartheta )$):

16 $$\hat{\varPhi }=\frac{{\omega }^{2}\left({\omega }_{0}^{2}X/g\right)}{\left({\omega }_{0}^{2}-{\omega }^{2}\right)+i2\hat{\zeta }{\omega }_{0}\omega },$$

$$\hat{I}=\frac{\hat{V}}{Z}=-{{\!\,}^{{EM}}\alpha }_{0}\frac{1}{Z+{Z}_{0}}i\omega \widehat{\varPhi };$$

$$\left\langle P\right\rangle =\frac{1}{T}{\int }_{0}^{T}{Z}^{{\prime} }{I}^{2}{dt}=\frac{1}{2}\frac{{\!\,}^{{EM}}\alpha _{0}^{2}}{{R}_{0}}\frac{{Z}_{0}^{{\prime} }{Z}^{{\prime} }}{{\left|Z+{Z}_{0}\right|}^{2}}{\omega }^{2}{\left|\widehat{\varPhi }\right|}^{2};$$

$$\eta =\frac{\frac{1}{2}\frac{{\!\,}^{{EM}}\alpha _{0}^{2}}{{R}_{0}}\frac{{Z}_{0}^{{\prime} }{Z}^{{\prime} }}{{\left|Z\, +\, {Z}_{0}\right|}^{2}}{\omega }^{2}{\left|\widehat{\varPhi }\right|}^{2}}{\frac{1}{2}\left(\frac{{\!\,}^{{EM}}\alpha _{0}^{2}}{{R}_{0}}\frac{{Z}_{0}^{{\prime} }\left({Z}^{{\prime} }+{Z}_{0}^{{\prime} }\right)}{{\left|Z\, +\, {Z}_{0}\right|}^{2}}+c\right){\omega }^{2}{\left|\widehat{\varPhi }\right|}^{2}-\frac{1}{T}{\int }_{0}^{T}{W}^{{Air}\to C}{dt}},$$

with $\widehat{\zeta }={\widehat{c}}_{\varPhi }/2{\omega }_{0}{I}_{1}$, ${\widehat{c}}_{\varPhi }=c+{\!\,}^{{EM}}\alpha _{0}^{2}/\left(Z+{Z}_{0}\right)$ and ${Z}_{0}={R}_{0}+i{L}_{0}$, and which has a known analytical solution that describes a linear resonant behavior. At low frequencies, the angle displacement increases with the square of the input frequency attaining a maximum peak value at a frequency close to $\omega ={\omega }_{0}$. At frequencies much larger than the natural frequency the angular displacement tends towards ${\omega }_{0}^{2}X/g$ ~ 11°. The output voltage and power follow a similar trend. The equivalent electrical circuit of the harvester in this approximation consists of a voltage source with output ${\widehat{V}}_{{OC}}={{{\mathrm{lim}}}}_{Z\to \infty }\widehat{V}$ in series with an equivalent frequency-dependent complex impedance ${Z}_{{Eq}}={\widehat{V}}_{{OC}}/{\widehat{I}}_{{SC}}$. The optimal load impedance for a maximum power output matches the complex conjugate of the equivalent internal impedance ($Z={Z}_{{Eq}}^{* }$; ${Z}_{{Eq}}={Z}_{0}+i(\omega /m){\!\,}^{{EM}}\alpha _{0}^{2}/[{\omega }_{0}^{2}-{\omega }^{2}+i(\omega /m)c]$), or $R=|{Z}_{{Eq}}|$ in the case of a purely resistive load.

**Fig. 7:**
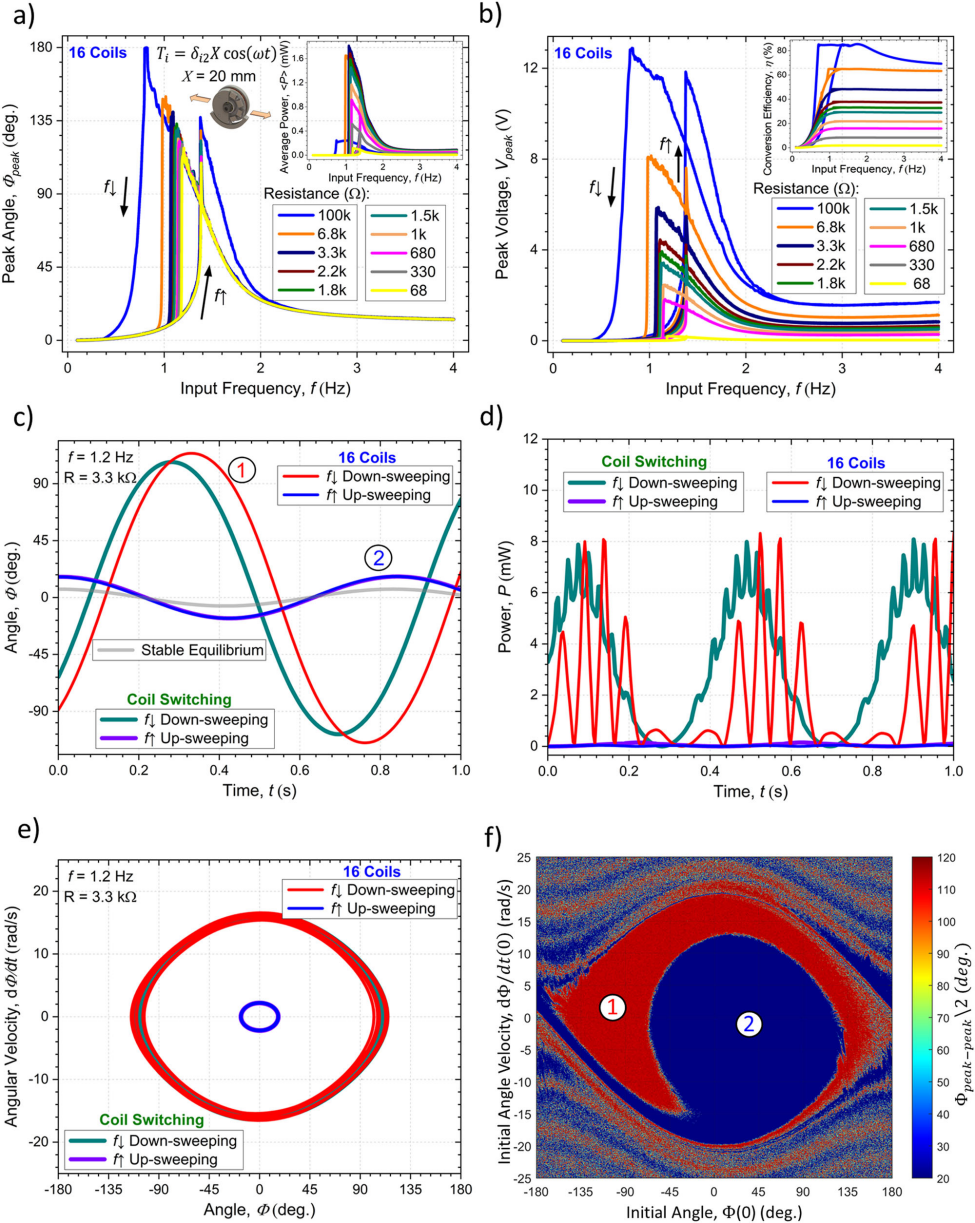
Calculated dynamic behavior of the harvester under the effects of an applied horizontal harmonic translational force with amplitude *X*=20 mm, multiple load resistances and an increasing and decreasing frequency. **a** Peak angular displacement of the rotator (average power in the inset), and **b**. Peak output voltage (energy conversion efficiency in the inset) as a function of the frequency of excitation in the system with 16 active coils. Time response of the **c**. Angular displacement, and **d**. Output power in the systems with 16 permanently active coils and with the coils switching architecture, with a load resistance of $R$ = 3.3 kΩ and frequency of $f$ = 1.2 Hz, initial conditions following a frequency up-sweeping or down-sweeping and corresponding **e**. Phase paths, and **f**. Basin of attraction of the system.

The more general Eq. (15) was solved numerically using a Runge-Kutta method (Matlab’s ode45 solver), low-frequency approximation ($I\, \approx \, - \!\left[{\!\,}^{{EM}}\alpha /\left(R+{R}_{0}\right)\right]\dot{\varPhi }$), resistive load ($V$ = ${RI}$) and the parameters of the fabricated EMG listed in Table 1, as well as a friction torque of the form: $-c\dot{\varPhi }=-{c}_{{vis}}\dot{\varPhi }-{c}_{{drag}}{{{\rm{sign}}}}(\dot{\varPhi })$, with viscous drag constant ${c}_{{vis}}$ = 2.${I}_{1}$ mN.m.s^-1^ and Coulomb dry friction constant ${c}_{{drag}}$ = 1.${I}_{1}$ mN.m. These damping factors have been obtained by regression analysis after fitting with experimentally obtained results. The frequency response of the system with 16 permanently active coils is depicted in Fig. 7a and b showing a characteristic hysteretic output with the non-linear resonant steady state overhanging to the lower frequencies when sweeping the input frequency in a downward direction. As the load resistance increases the maximum peak angle that is attained decreases due to the associated increase in the EM damping factor. The discrete Fourier transform of the output angle has a main component with the same frequency as the input frequency while that of the voltage has one to four times this frequency due to the multiple peaks of the EM coupling factor previously depicted in Fig. 4f. The displacement angle also tends to be in phase with the input excitation at low frequencies ($\omega \, \ll \, {\omega }_{0}$) and lag up to 180° at high frequencies ($\omega \, \gg \, {\omega }_{0}$), in agreement with the linear solution in Eq. (16). A maximum output average power of ~1.8 mW is obtained for a matching load of 3.3 kΩ, while a conversion efficiency of up to ~80% can be obtained at a load of 100 kΩ. We note that the coil switching architecture permits increasing the average power to 3.1 mW and efficiency to ~90% under the same load conditions. The time response of the harvester with 16 permanently active coils and the implemented coil switching approach is illustrated in Fig. 7c–e, with a load resistance of $R$ = 3.3 kΩ and frequency of $f$ = 1.2 Hz attained after an up-sweeping or down-sweeping. This shows that, depending on the system’s initial conditions, under these excitation parameters it has two different steady-state outputs, one non-linear resonant one with a large angular amplitude attained when down-sweeping the frequency and one non-resonant state with lower amplitude obtained when up-sweeping the frequency. Figure 7e shows the corresponding phase paths of these two limit cycles as the system follows the angle for the minimum of effective potential energy with a certain phase lag as it shifts with time. The calculated basin of attraction of the system, showing the steady-state io which it evolves depending on its initial conditions, is illustrated in Fig. 7f. This indicates that the high amplitude non-linear resonant state can be achieved for initial conditions of sufficiently large angle or angular velocity and at a given phase relative to the input translation which are obtained more easily during the frequency down-sweeping process. At frequencies sufficiently larger or smaller than the natural frequency of the harvester and for smaller input amplitudes or larger damping factors, there is only a single non-resonant limit cycle.

**Fig. 8:**
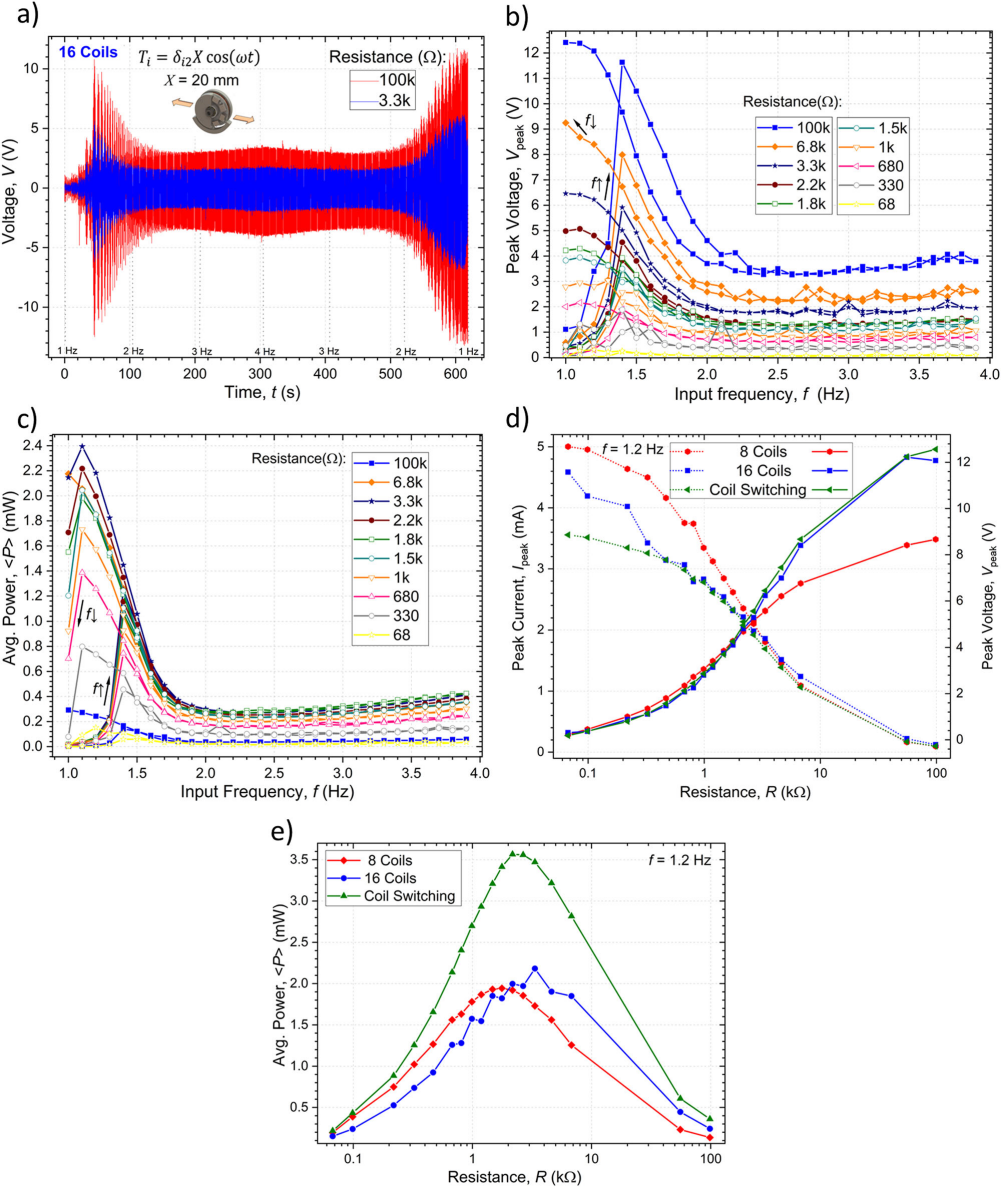
Electric characterization related to energy harvesting using harmonic translational excitations. **a** Measured voltage as a function of time for input horizontal translational harmonic oscillations with increasing and subsequent decreasing frequency increments from 1 Hz to 4 Hz. **b** Frequency response of the peak voltage in the pendulum system for different loads. **c** Average power as a function of the input frequency. **d** Experimental peak voltage for 16 coils and theoretical peak voltage for 8 coils and system using coil switching (right scale), and experimental peak current for 16 coils and theoretical peak current for 8 coils and system using coil switching (left scale). **e** Results of the average output power as a function of resistance for a resonance frequency of 1.2 Hz for experimental tests using 16 coils and theoretical tests using 8 coils and coil switching.

Output voltage measurements were performed using translational movements, by increasing/decreasing frequency in the range between 0.1 Hz and 4 Hz. Figure 8a shows the optimal performance of the non-adaptive 16-coil harvester, which was achieved by using a load of 3.3 kΩ. The resonant behavior clearly emerges, whose spectrum range mainly occurs between 1.1 Hz and 1.4 Hz (Fig. 8b an c). Optimal peak electric currents of 2.17 mA (for 2.2 kΩ), 1.85 mA (for 3.3 kΩ), and 2.32 mA (for 1.8 kΩ) were found for the adaptive harvester, non-adaptive 16-coil harvester and non-adaptive 8-coil harvester, respectively, even though maximum currents of 3.55 mA, 4.58 mA and 5 mA (Fig. 8d). Concerning average power (Figs. 8e), 3.56 mW (for 2.2 kΩ) was achieved for the adaptive harvester, which corresponds to a 63.3% increase compared to the non-adaptive 16-coil harvester (2.18 mW @ 3.3 kΩ) and 83.5% increase compared to the non-adaptive 8-coil harvester (1.94 mW @ 1.8 kΩ).

#### Harmonic swaying excitation

As illustrated in Fig. 2c, we also considered the case of applied rotations to the stator by an angle $\varphi$ around the axis of symmetry of the harvester with: ${\omega }_{I}={\delta }_{I1}\dot{\varphi }$ and with a pendulum-like translation of: ${T}_{i}={l}_{0}(0,\sin \varphi ,-\cos \varphi )$, with ${l}_{0}$ the constant distance from the rotation pivot point to the center of the stator. With these parameters, Eq. (12) and the circuit Eq. (9) can be simplified into a subsystem of just two differential equations:

17 $${I}_{1}\ddot{\varPhi }= - \! {I}_{1}\ddot{\varphi }-m{X}^{{CM}}\left[g\sin \left(\varPhi +\varphi \right)+{l}_{0}{\dot{\varphi }}^{2}\sin \left(\varPhi \right)\right.\\ \left.+\, {l}_{0}\ddot{\varphi }\cos \left(\varPhi \right)\right]-c\dot{\varPhi }+{\!\,}^{{EM}}\alpha \, I,$$

which describes the dynamics of the harvester and can be solved for the angle $\varPhi$ and current $I$ for any input time-changing angle $\varphi$ and with prescribed initial conditions. Equation (17) corresponds to the equation of a forced non-linear oscillator and could also be obtained through the use of Lagrangian mechanics. The non-linear restoring force has the form: $m{X}^{{CM}}g\sin \left(\varphi +\varPhi \right)$, which corresponds to an equivalent elastic stiffness constant $k=m{X}^{{CM}}\left[g\cos \left(\varphi +\varPhi \right)+{l}_{0}{\dot{\varphi }}^{2}\cos \left(\varPhi \right)-{l}_{0}\ddot{\varphi }\sin \left(\varPhi \right)\right]$. There are also centrifugal torques in the form of: $m{X}^{{CM}}{l}_{0}\sin \left(\varPhi \right){\dot{\varphi }}^{2}$, which increases with the exciting angular velocity $\dot{\varphi }$ and tends to pull the angle towards the $\varPhi =0$ position and thus the eccentric mass further away from the pivot point, and Euler torques in the form of: $m{X}^{{CM}}{l}_{0}\cos \left(\varPhi \right)\ddot{\varphi }$, e.g. responsible for driving the eccentric mass forward after bringing the stator suddenly to a full stop.

In the case of the simple swaying motion, a harmonic angular excitation of the form: $\varphi =-\varDelta \varphi \cos (\omega t)$, with a constant amplitude $\varDelta \varphi$ = 10°, rotation rate $\omega$ and ${l}_{0}$ = 140 mm, was applied to the harvester. In the regime of low-frequency operation ($\omega \, \ll \, {\omega }_{0}$) and low output angular displacements the equation of motion has an approximate analytic harmonic solution similar to that in Eq. (16) with the transformation ${\omega }_{0}^{2}X/g\to \varDelta \varphi$. At these low frequencies, the angle of stable equilibrium is simply: ${\varPhi }_{0}=\vartheta +\pi /2=-\varphi =\varDelta \varphi \cos (\omega t)$, and the eccentric mass tends to be pulled towards its lowest position thus minimizing the gravitational potential energy. At high frequencies ($\omega \, \gg \, {\omega }_{0}$), and with relatively small input angle amplitudes $\varDelta \varphi$, the equilibrium position ${\varPhi }_{0}$ oscillates between $-(\pi /2+\theta )$ and $(\pi /2+\theta )$ as $\omega t$ changes between $0$ and $\pi$, with $\theta =\pi /2-{\cos }^{-1}(1/\beta )$; $\beta =m{X}^{{CM}}{l}_{0}/{I}_{1}$. There might not exist equilibrium states during part of the period when ${l}_{0} \, < \, {I}_{1}/m{X}^{{CM}} \sim 103\,{{{\rm{mm}}}}$. In the limit, the peak angle of the output tends approximately towards $(1+\beta )\varDelta \varphi$ ~ 24°. Equation (17) in its most general form does not have analytic solutions and therefore must be solved numerically. Qualitatively this yields a behavior similar to that studied in the previous harmonic translation excitation.

**Fig. 9:**
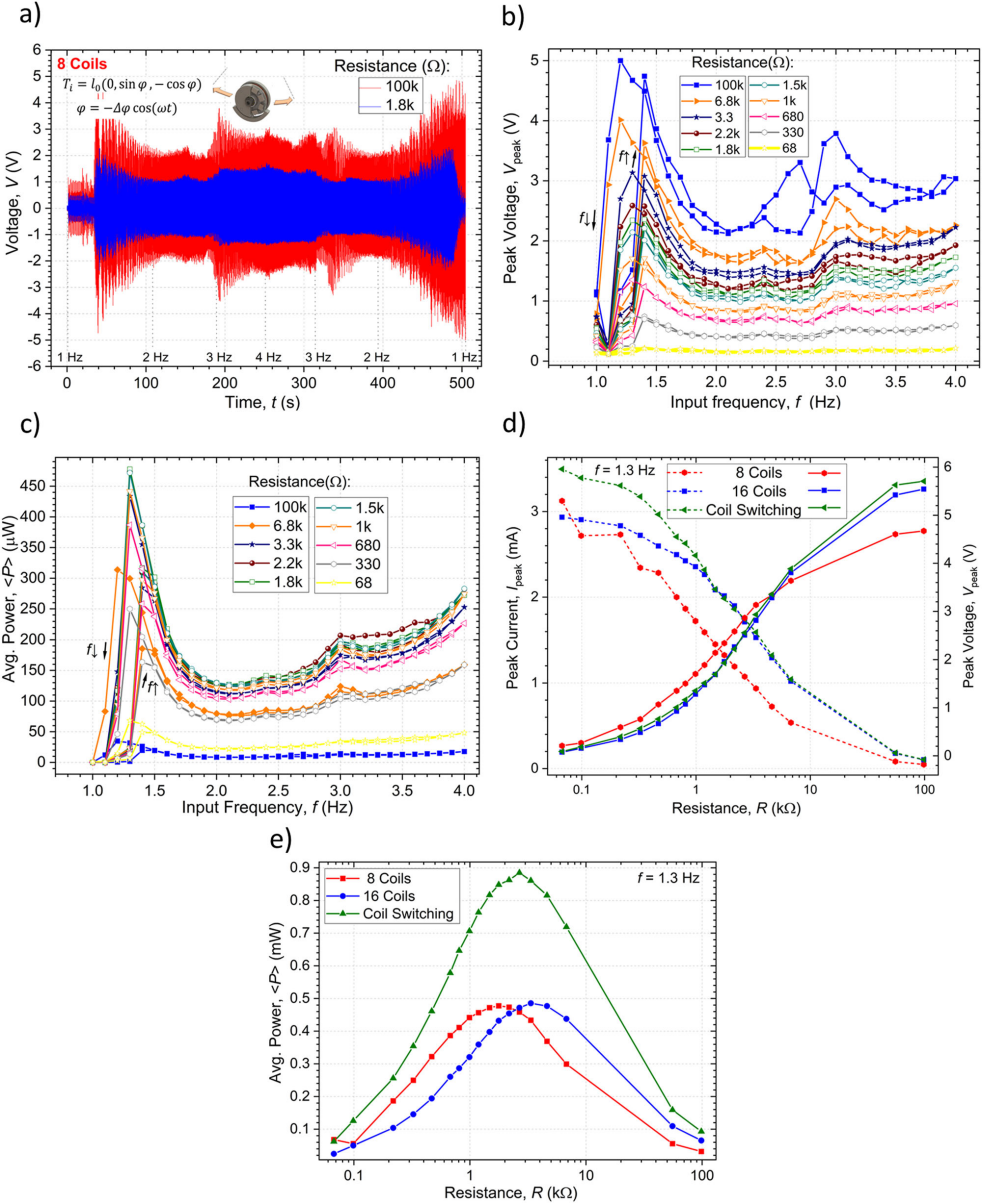
Electric characterization related to energy harvesting using harmonic pendulum excitations. **a** Measured voltage as a function of time for input sinusoidal pendulum oscillations with increasing increments from 1 Hz to 4 Hz and subsequent decrease. **b** Frequency response of the peak voltage in the pendulum system for different loads. **c** Average power as a function of the input frequency. **d** Experimental peak voltage for 16 coils and theoretical peak voltage for 8 coils and system using coil switching (right scale), and experimental peak current for 8 coils and theoretical peak current for 16 coils and the system using coil switching (left scale). **e** Results of the average output power as a function of resistance for a resonance frequency of 1.3 Hz for experimental tests using 8 coils and theoretical tests using 16 coils and coil switching.

Output voltage measurements were performed using swaying movements, by increasing/decreasing the frequency in the range between 0.1 Hz and 4 Hz. This is suitable to analyze the harvester’s performance for various applications, including for biomedical devices or sea wave energy systems. Figure 9a shows the optimal performance of the non-adaptive 8-coil harvester, which was achieved by using a load of 1.8 kΩ. The nonlinear resonant behavior clearly emerges, whose spectrum range mainly occurs between 1.2 Hz and 1.4 Hz (Fig. 9b and c). Optimal electric currents of 1.75 mA (for 2.7 kΩ), 1.5 mA (for 3.3 kΩ), and 1.3 mA (for 1.8 kΩ) were found for the adaptive harvester, non-adaptive 16-coil harvester and non-adaptive 8-coil harvester, respectively, even though maximum currents of 3.5 mA, 2.9 mA and 3.1 mA (Fig. 9d). These results show the complexity of the pendulum system behavior in response to these mechanical excitation scenarios, with the difference between the resonant frequencies slightly increasing with an increasing load for the two discontinuity jumps. The identification of this nonlinear resonance is crucial to identify the peculiar characteristics of the system at specific frequencies, which may have important implications in the design and optimization of systems involving pendulum motion. Optimal electric currents of 1.75 mA (for 2.7 kΩ), 1.53 mA (for 3.3 kΩ), and 1.32 mA (for 1.8 kΩ) were found for the adaptive harvester, non-adaptive 16-coil harvester and non-adaptive 8-coil harvester, respectively, even though maximum currents of 3.49 mA, 2.93 mA and 3.12 mA (Fig. 9d). Concerning average power (Fig. 9e), 0.88 mW (for 2.7 kΩ) was achieved for the adaptive harvester, which corresponds to a 79.5% increase compared to the non-adaptive 16-coil harvester (0.49 mW @ 3.3 kΩ) and 87.2% increase as compared to the non-adaptive 8-coil harvester (0.47 mW @ 1.8 kΩ).

## Conclusions

This study presents the concept of a rotating harvester with high energy conversion efficiency, characterized by the implementation of a dynamic coil switching mechanism that enables real-time optimization of the coil connection configuration during operation. This work extensively analyzes the frequency response of a rotating electromagnetic harvester using two different excitation methods: (i) simple axial rotation; and (ii) general 3D motion of the stator, including a harmonic horizontal translation and swaying movement of a moving arm. These tests were effective in determining the electromagnetic coefficient and resonance frequency, as well as in validating the practical applicability of this technology in real-world scenarios. The strongly non-linear dynamics of the oscillator was studied, including the identification of the presence of one stable and one unstable equilibrium configuration of the system or the entire absence of such configurations depending on the time-changing characteristics of the input translations and rotations. The system was shown to possess a softening stiffness with a resonant hysteretic frequency response overhanging to the lower frequencies when down-seeping the input frequency. For frequencies close to the natural frequency of the harvester, two steady-state limit cycles were observed, one high amplitude non-linear resonant state and one low amplitude non-resonant state, the first of which was attained under initial conditions of sufficiently large angular displacement or angular velocity obtained, for example, during frequency down-sweeping.

Various coil configurations of the harvester were tested. The studied electric architectures of the RH included: (i) the simultaneous operation of 16 permanently active coils; (ii) 8 permanently active odd numbered coils; (iii) an adaptive system comprising real-time dynamic switching of the two groups of even and odd numbered coils. The EMG system operating under a pendulum-like motion showed a non-linear and resonant response, characterized by a softening stiffness and a hysteretic frequency response overhanging to the lower frequencies. The results of the experimental axial rotation tests at a frequency of 5 Hz (300 rpm), resulted in peak voltages of 6.01 V for the 16 coil-architecture and 3.78 V for the 8 coil-architecture, and peak currents of 1.79 mA and 2.14 mA, respectively. Under ideal load conditions, the 16 coil-architecture was able to harvest an average output power up to 5.1 mW, but the 8 coil-architecture only reached 4 mW. Remarkably, the dynamic switching architecture allowed significant increase in the output average power, which reached 10 mW, and the estimated energy conversion efficiency was also enhanced from ~80% to 90%.

In the general 3D mechanical excitations tests for harmonic translation and harmonic swaying excitations, a noticeable percentage increase of 63.3% and 79.6%, respectively, was found in the average power generated when comparing the adaptive harvester and the non-adaptive 16-coil harvester. Using the coil-switching strategy, an average power density of 836.1 ${{{\rm{W}}}}{{{{\rm{m}}}}}^{-3}$ was obtained with simple rotations, and 583.6 ${{{\rm{W}}}}{{{{\rm{m}}}}}^{-3}$ and 144.3 ${{{\rm{W}}}}{{{{\rm{m}}}}}^{-3}$ for harmonic translation and harmonic swaying excitations. These results, even using a non-complex rotational electromagnetic architecture, provided higher power densities than those usually obtained by triboelectric and piezoelectric harvester^23,25,79^. These are the complex architectures developed by An et al.^42^ Liang et al.^44^ Abdelkareem et al.^43^ and Li et al.^80^ which reported the following peak power densities: 12.4 ${{{\rm{W}}}}{{{{\rm{m}}}}}^{-3}$, 4.8 ${{{\rm{W}}}}{{{{\rm{m}}}}}^{-3}$, 50 ${{{\rm{W}}}}{{{{\rm{m}}}}}^{-3}$, and 18.2 m${{{\rm{W}}}}{{{{\rm{m}}}}}^{-3},{{{\rm{respectively}}}}$. This demonstrates that, even when comparing average power densities from our adaptive rotational EMG with peak power densities from triboelectric and piezoelectric harvesters, our adaptive harvester can provide gains significantly higher even using a simpler architecture without modifying the harvester’s design. This concept can be used in more complex architectures, including hybrids, to ensure outstanding efficiencies for a wide range of mechanical excitation dynamics. Although rotational electromagnetic generation is found as a viable alternative to triboelectric and piezoelectric generations, many applications may benefit from self-powering based on hybrid technologies, namely when very high voltages and low currents are required (e.g. to supply capacitive sensing and therapeutic systems^81,82^). Furthermore, the use of adaptive rotational hybrid harvesters can provide both high electric currents and high voltages and allow redundant powering. It is also worth highlighting the ability of our adaptive harvester to be engineered according to both small-scale and large-scale architectures, holding the potential to replace piezoelectric and triboelectric harvesters even in micro-scale applications. Indeed, even though electromagnetic harvesters are usually considered ineffective in small-scale powering^25^, advanced methods have been developed to manufacture and optimize micro-sized coils^83–85^, which can pave the way towards the development of ever smaller adaptive rotational systems^86^. Finally, more complex controllers will most likely be required (e.g., predictive control) as the complexity of harvesters’ architectures increases, as well as the nonlinearity of mechanical excitations is higher. The development of Artificial Intelligence algorithms can be used to predict mechanical excitation patterns so that the harvested energy can be maximized over specific time horizons.

In summary, the important findings reported in this work can be further mastered and applied to advanced energy harvesting solutions via:

1. Optimization of the shape of the magnets and coils to maximize the power factor of the associated EM coupling through the calculus of variations.
2. Development of other adaptive rotational harvesters based on autonomous coil switching mechanisms, as well as other multi-dimensional harvesters. Using our adaptive technology, many other EMGs will most likely be able to significantly maximize both the output power and efficiency for different mechanical configurations and input vibrational mechanical characteristics.
3. Development of more complex circuitry, capable of switching the coils between parallel and series configurations to allow optimized tuning of the internal impedance characteristics.

## Methods

### Mechanical excitation apparatus

The digital input/output channels of the dSPACE DS1104 DSP board were integrated with the Matlab (v. 9.4, MathWorks) and Simulink (v. 9.1, MathWorks) development environments, using the Real-Time Workshop (v. 5.6, MathWorks) and Real-Time Interface (v. 7.12, dSPACE) tools. Two software applications were developed in the ControlDesk software (v. 7.0, dSPACE) to interact with a real-time system. These applications allowed to carry out all the mechanical excitations using 2 different structures containing: (1) a stepper motor driver (DM856) and a NEMA 34 stepper motor (ACT 34HS1456); and (2) an AC motor (W21 90S, WEG) with frequency inverter (EFC3610-1 K50, Bosch). Two different test scenarios were used: (i) mechanical axial rotations: the rotator was directly coupled to the shaft of the NEMA motor, such that it could be rotated at a given speed relative to the fixed stator (Fig. 1d); (ii) general 3D movements, including a 40 mm horizontal harmonic translation of the stator, using a sliding crank mechanism driven by the AC motor (Fig. 1e) (see Supplementary Movie 1) and a mechanical swaying pendulum movement: a 140 mm long movable arm was perpendicularly attached to the motor shaft so that pendulum movements are delivered to the stator (Fig. 1f) (see Supplementary Movie 2).

### Experimental testing

The frequency response of the output voltage provided by the RH was used to measure the values of the electromechanical (EM) coefficient and its resonance frequency in the general 3D mechanical excitation tests. These measurements were performed under two different conditions. In the first input scenario, an axial rotation was applied to the rotator coupled to the motor, within a frequency range between 0 Hz and 5 Hz (300 rpm), and with increasing frequency steps of 0.5 Hz. The second input scenario for general 3D mechanical excitations was characterized by applying a sinusoidal excitation, translational or swaying pendulum, with increasing followed by decreasing frequency steps of 0.1 Hz, between 1 Hz and 4 Hz. Both tests were performed with load resistances ranging from 47 Ω to 10 kΩ. In addition to the tests performed with all coil groups simultaneously connected (16-coil architecture), experiments were also conducted to evaluate the performance of different coil configurations, namely the 8-coil architecture (connection of only one group of even-numbered coils). These were performed to compare the adaptive harvester’s performance when it is connected with 16 or 8 coils, as required by the switching system.

### Supplementary information

Description of Additional Supplementary Files

Supplementary Movie 1

Supplementary Movie 2

Supplementary Movie 3

## References

[1] Lv, H. et al. A flexible electromagnetic wave-electricity harvester. *Nat. Commun.* **12**, 834 (2021). 33547310 10.1038/s41467-021-21103-9 PMC7864982

[2] Zhang, C. et al. Conjunction of triboelectric nanogenerator with induction coils as wireless power sources and self-powered wireless sensors. *Nat. Commun.* **11**, 58 (2020). 31896757 10.1038/s41467-019-13653-w PMC6940365

[3] Calautit, K., Nasir, D. S. N. M. & Hughes, B. R. Low power energy harvesting systems: State of the art and future challenges. *Renew. Sust. Energ. Rev.* **147**, 111230 (2021). 10.1016/j.rser.2021.111230

[4] Mitcheson, P. D., Green, T. C., Yeatman, E. M. & Holmes, A. S. Architectures for vibration-driven micropower generators. *J. Microelectromech. Syst.* **13**, 429 (2004). 10.1109/JMEMS.2004.830151

[5] Kim, J. et al. Wearable smart sensor systems integrated on soft contact lenses for wireless ocular diagnostics. *Nat. Commun.* **8**, 14997 (2017). 28447604 10.1038/ncomms14997 PMC5414034

[6] Sharma, R. et al. Electrically connected spin-torque oscillators array for 2.4 GHz WiFi band transmission and energy harvesting. *Nat. Commun.* **12**, 2924 (2021). 34006830 10.1038/s41467-021-23181-1 PMC8131736

[7] Dunn, J. & Kidzinski, L. Wearable sensors enable personalized predictions of clinical laboratory measurements. *Nat. Med.* **27**, 1105 (2021). 34031607 10.1038/s41591-021-01339-0 PMC8293303

[8] Soares dos Santos, M. P. et al. Towards an effective sensing technology to monitor micro-scale interface loosening of bioelectronic implants. *Sci. Rep.* **11**, 3449 (2021). 33568680 10.1038/s41598-021-82589-3 PMC7876021

[9] Ausra, J. et al. Wireless battery free fully implantable multimodal recording and neuromodulation tools for songbirds. *Nat. Commun.* **12**, 1968 (2021). 33785751 10.1038/s41467-021-22138-8 PMC8009877

[10] Peres, I., Rolo, P. & Soares Dos Santos, M. P. Multifunctional Smart Bone Implants: Fiction or Future?-A New Perspective. *Front Bioeng. Biotechnol.* **10**, 912081 (2022). 35757794 10.3389/fbioe.2022.912081 PMC9216553

[11] Soares dos Santos, M. P. et al. Capacitive technologies for highly controlled and personalized electrical stimulation by implantable biomedical systems. *Sci. Rep.* **9**, 5001 (2019). 30899061 10.1038/s41598-019-41540-3 PMC6428833

[12] Kim, C. Y. et al. Soft subdermal implant capable of wireless battery charging and programmable controls for applications in optogenetics. *Nat. Commun.* **12**, 535 (2021). 33483493 10.1038/s41467-020-20803-y PMC7822865

[13] Ali, A. et al. A review of energy harvesting from regenerative shock absorber from 2000 to 2021: advancements, emerging applications, and technical challenges. *Environ. Sci. Pollut. Res. Int.* **30**, 5371 (2023). 36414897 10.1007/s11356-022-24170-7

[14] Panda, S. et al. Hybrid Nanogenerators for Ocean Energy Harvesting: Mechanisms, Designs, and Applications. *Small* **19**, 2300847 (2023). 10.1002/smll.202300847 36929123

[15] Ben Amar, A., Kouki, A. B. & Cao, H. Power Approaches for Implantable Medical Devices. *Sensors (Basel)* **15**, 28889 (2015). 26580626 10.3390/s151128889 PMC4701313

[16] Joung, Y. H. Development of implantable medical devices: from an engineering perspective. *Int. Neurourol. J.* **17**, 98 (2013). 24143287 10.5213/inj.2013.17.3.98 PMC3797898

[17] Soares dos Santos, M. P., Ferreira, J. A. F., Ramos, A. & Simões, J. A. O. Active orthopaedic implants: Towards optimality. *J. Franklin Inst.* **352**, 813 (2015). 10.1016/j.jfranklin.2014.11.005

[18] Yu, J. et al. Contact-electrification-activated artificial afferents at femtojoule energy. *Nat. Commun.* **12**, 1581 (2021). 33707420 10.1038/s41467-021-21890-1 PMC7952391

[19] Liu, X., Zhang, M., Richardson, A. G., Lucas, T. H. & Spiegel, J. V. D. Design of a Closed-Loop, Bidirectional Brain Machine Interface System With Energy Efficient Neural Feature Extraction and PID Control. *IEEE Trans. Biomed. Circuits Syst.* **11**, 729 (2017). 28029630 10.1109/TBCAS.2016.2622738

[20] Vidal, J. V. et al. Automated electromagnetic generator with self-adaptive structure by coil switching. *Appl. Energy* **325**, 119802 (2022). 10.1016/j.apenergy.2022.119802

[21] Carneiro, P. M. R. et al. Instrumented electromagnetic generator: Optimized performance by automatic self-adaptation of the generator structure. *Mech. Syst. Signal Process.* **171**, 108898 (2022). 10.1016/j.ymssp.2022.108898

[22] Bai, Y., Feng, H. & Li, Z. Theory and applications of high-voltage triboelectric nanogenerators. *Cell. Rep. Phys. Sci.* **3**, 101108 (2022). 10.1016/j.xcrp.2022.101108

[23] Rodrigues, C. et al. Emerging triboelectric nanogenerators for ocean wave energy harvesting: state of the art and future perspectives. *Energy Environ. Sci.* **13**, 2657 (2020). 10.1039/D0EE01258K

[24] Pan, M. et al. Triboelectric and Piezoelectric Nanogenerators for Future Soft Robots and Machines. *iScience* **23**, 101682 (2020). 33163937 10.1016/j.isci.2020.101682 PMC7607424

[25] Li, Z., Roscow, J., Khanbareh, H., Haswell, G. & Bowen, C. “Energy Harvesting from Water Flow by Using Piezoelectric Materials”. *Adv. Energy Sustainability Res*. **5**, 2300235 (2024). 10.1002/aesr.202300235

[26] Vidal, J. V. et al. Low-Frequency Vibration Energy Harvesting With Bidomain LiNbO3 Single Crystals. *IEEE Trans. Ultrason. Ferroelectr. Freq. Control* **66**, 1480 (2019). 30990180 10.1109/TUFFC.2019.2908396

[27] Vidal, J. V. et al. Dual vibration and magnetic energy harvesting with bidomain LiNbO3 based composite. *IEEE Trans. Ultrason. Ferroelectr. Freq. Control* **67**, 1219 (2020). 31985416 10.1109/TUFFC.2020.2967842

[28] Xiao, T. X. et al. Spherical Triboelectric Nanogenerators Based on Spring-Assisted Multilayered Structure for Efficient Water Wave Energy Harvesting. *Adv. Funct. Mater.* **28**, 1802634 (2018). 10.1002/adfm.201802634

[29] Fu, H., Jiang, J., Hu, S., Rao, J. & Theodossiades, S. A multi-stable ultra-low frequency energy harvester using a nonlinear pendulum and piezoelectric transduction for self-powered sensing. *Mech. Syst. Signal Process.* **189**, 110034 (2023). 10.1016/j.ymssp.2022.110034

[30] Kang, M. et al. Triboelectric neurostimulator for physiological modulation of leg muscle. *Nano Energy* **103**, 107861 (2022). 10.1016/j.nanoen.2022.107861

[31] Kim, H. S., Kim, J.-H. & Kim, J. A review of piezoelectric energy harvesting based on vibration. *Int. J. Precis. Eng. Man.* **12**, 1129 (2011). 10.1007/s12541-011-0151-3

[32] Covaci, C. & Gontean, A. Piezoelectric Energy Harvesting Solutions: A Review. *Sensors* **20**, 3512 (2020). 32575888 10.3390/s20123512 PMC7349337

[33] Zheng, X. et al. A review of piezoelectric energy harvesters for harvesting wind energy. *Sens. Actuators A: Phys*. **352**, 114190 (2023). 10.1016/j.sna.2023.114190

[34] Wang, W. et al. Triboelectric nanogenerators: the beginning of blue dream. *Front. Chem. Sci. Eng.* **17**, 635 (2023). 10.1007/s11705-022-2271-y

[35] Du, T. et al. Recent Advances in Mechanical Vibration Energy Harvesters Based on Triboelectric Nanogenerators. *Small* **19**, 2300401 (2023). 10.1002/smll.202300401 36840670

[36] Vidal, J. V., Slabov, V., Kholkin, A. L. & dos Santos, M. P. S. Hybrid Triboelectric-Electromagnetic Nanogenerators for Mechanical Energy Harvesting: A Review. *Nano-Micro Lett*. **13**, 199 (2021). 10.1007/s40820-021-00713-4 PMC8452823 34542731

[37] Sezer, N. & Koç, M. A comprehensive review on the state-of-the-art of piezoelectric energy harvesting. *Nano Energy* **80**, 105567 (2021). 10.1016/j.nanoen.2020.105567

[38] Gielen, D., Boshell, F. & Saygin, D. Climate and energy challenges for materials science. *Nat. Mater.* **15**, 117 (2016). 26796720 10.1038/nmat4545

[39] Yin, J., Molini, A. & Porporato, A. Impacts of solar intermittency on future photovoltaic reliability. *Nat. Commun.* **11**, 4781 (2020). 32963258 10.1038/s41467-020-18602-6 PMC7508863

[40] Zhang, Y., Zhao, Y., Sun, W. & Li, J. Ocean wave energy converters: Technical principle, device realization, and performance evaluation. *Renew. Sust. Energ. Rev.* **141**, 110764 (2021). 10.1016/j.rser.2021.110764

[41] Barnier, B. et al. Modelling the impact of flow-driven turbine power plants on great wind-driven ocean currents and the assessment of their energy potential. *Nat. Energy* **5**, 240 (2020). 10.1038/s41560-020-0580-2

[42] An, J., Wang, Z. M., Jiang, T., Liang, X. & Wang, Z. L. Whirling-Folded Triboelectric Nanogenerator with High Average Power for Water Wave Energy Harvesting. *Adv. Funct. Mater.* **29**, 1904867 (2019). 10.1002/adfm.201904867

[43] Abdelkareem, M. A. A., Jing, X., Eldaly, A. B. M. & Choy, Y. 3-DOF X-structured piezoelectric harvesters for multidirectional low-frequency vibration energy harvesting. *Mech. Syst. Signal Process.* **200**, 110616 (2023). 10.1016/j.ymssp.2023.110616

[44] Liang, X. et al. Spherical triboelectric nanogenerator integrated with power management module for harvesting multidirectional water wave energy. *Energy Environ. Sci.* **13**, 277 (2020). 10.1039/C9EE03258D

[45] Elliott, D. A balancing act for renewables. *Nat. Energy* **1**, 15003 (2016). 10.1038/nenergy.2015.3

[46] Soares dos Santos, M. P. et al. Magnetic levitation-based electromagnetic energy harvesting: a semi-analytical non-linear model for energy transduction. *Sci. Rep.* **6**, 18579 (2016). 26725842 10.1038/srep18579 PMC4698582

[47] Carneiro, P. et al. Electromagnetic energy harvesting using magnetic levitation architectures: A review. *Appl. Energy* **260**, 114191 (2020). 10.1016/j.apenergy.2019.114191

[48] Lacks, D. J. & Shinbrot, T. Long-standing and unresolved issues in triboelectric charging. *Nat. Rev. Chem.* **3**, 465 (2019). 10.1038/s41570-019-0115-1

[49] Yan, Y. et al. Triboelectric-electromagnetic hybrid generator with swing-blade structures for effectively harvesting distributed wind energy in urban environments. *Nano Res.* **16**, 11621 (2023). 10.1007/s12274-023-5691-1

[50] Koh, K. H. et al. A self-powered 3D activity inertial sensor using hybrid sensing mechanisms. *Nano Energy* **56**, 651 (2019). 10.1016/j.nanoen.2018.11.075

[51] Egbe, K.-J. I., Matin Nazar, A. & Jiao, P. Piezoelectric-triboelectric-electromagnetic Hybrid Rotational Energy Harvesters (H-REH).*Int. J. Mech* **235**, 107722 (2022). 10.1016/j.ijmecsci.2022.107722

[52] Peng, Y., Xu, W., Gong, Y., Peng, X. & Li, Z. Electromechanical coupling of a 3.88 W harvester with circumferential step-size field: modeling, validation and self-powered wearable applications. *Smart Mater. Struct.* **33**, 025039 (2024). 10.1088/1361-665X/ad1d72

[53] Rahman, M. T. et al. Biomechanical Energy-Driven Hybridized Generator as a Universal Portable Power Source for Smart/Wearable Electronics. *Adv. Energy Mater.* **10**, 1903663 (2020). 10.1002/aenm.201903663

[54] Zhou, X. et al. An ultra-compact lightweight electromagnetic generator enhanced with Halbach magnet array and printed triphase windings. *Appl. Energy* **353**, 122075 (2024). 10.1016/j.apenergy.2023.122075

[55] Fu, H. et al. Rotational energy harvesting for self-powered sensing. *Joule* **5**, 1074 (2021). 10.1016/j.joule.2021.03.006

[56] Berdy, D. F., Valentino, D. J. & Peroulis, D. Design and optimization of a magnetically sprung block magnet vibration energy harvester. *Sens. Actuators A: Phys* **218**, 69 (2014). 10.1016/j.sna.2014.06.011

[57] Halim, M. A. et al. An electromagnetic rotational energy harvester using sprung eccentric rotor, driven by pseudo-walking motion. *Applied Energy* **217**, 66 (2018). 10.1016/j.apenergy.2018.02.093

[58] Fan, K. et al. An eccentric mass-based rotational energy harvester for capturing ultralow-frequency mechanical energy. *Energy Convers. Manag.* **241**, 114301 (2021). 10.1016/j.enconman.2021.114301

[59] Zhang, H., Wu, X., Pan, Y., Azam, A. & Zhang, Z. A novel vibration energy harvester based on eccentric semicircular rotor for self-powered applications in wildlife monitoring. *Energy Convers. Manag.* **247**, 114674 (2021). 10.1016/j.enconman.2021.114674

[60] Li, R. et al. A rotational energy harvester with a semi-flexible one-way clutch for capturing low-frequency vibration energy. *Energy* **281**, 128266 (2023). 10.1016/j.energy.2023.128266

[61] Holmes, A. S., Guodong, H. & Pullen, K. R. Axial-flux permanent magnet machines for micropower generation. *J. Microelectromech. Syst.* **14**, 54 (2005). 10.1109/JMEMS.2004.839016

[62] He, J. et al. 3D full-space triboelectric-electromagnetic hybrid nanogenerator for high-efficient mechanical energy harvesting in vibration system. *Energy* **194**, 116871 (2020). 10.1016/j.energy.2019.116871

[63] Huo, S. et al. Dual-mode electromagnetic energy harvester by Halbach arrays. *Energy Convers. Manag.* **286**, 117038 (2023). 10.1016/j.enconman.2023.117038

[64] Xu, W., Wong, M.-C. & Hao, J. Strategies and progress on improving robustness and reliability of triboelectric nanogenerators. *Nano Energy* **55**, 203 (2019). 10.1016/j.nanoen.2018.10.073

[65] Lai, Z., Xu, J., Bowen, C. R. & Zhou, S. Self-powered and self-sensing devices based on human motion. *Joule* **6**, 1501 (2022). 10.1016/j.joule.2022.06.013

[66] Hassanpour Amiri, M. et al. Piezoelectric energy harvesters: a critical assessment and a standardized reporting of power-producing vibrational harvesters. *Nano Energy* **106**, 108073 (2023). 10.1016/j.nanoen.2022.108073

[67] Guo, S. et al. Mechanism, theory and application research of a rotating electromagnetic energy harvester suitable for multi-directional excitation. *J. Phys. D: Appl. Phys.* **55**, 085503 (2022). 10.1088/1361-6463/ac3582

[68] Wang, T. Pendulum-based vibration energy harvesting: Mechanisms, transducer integration, and applications. *Energy Convers. Manag.* **276**, 116469 (2023). 10.1016/j.enconman.2022.116469

[69] Wu, X. et al. Foundations of offshore wind turbines: A review. *Renew. Sust. Energ. Rev.* **104**, 379 (2019). 10.1016/j.rser.2019.01.012

[70] Hossain, M. M. & Ali, M. H. Future research directions for the wind turbine generator system. *Renew. Sust. Energ. Rev.* **49**, 481 (2015). 10.1016/j.rser.2015.04.126

[71] Yurchenko, D. & Alevras, P. Parametric pendulum based wave energy converter. *Mech. Syst. Signal Process.* **99**, 504 (2018). 10.1016/j.ymssp.2017.06.026

[72] Miao, G., Fang, S., Wang, S. & Zhou, S. A low-frequency rotational electromagnetic energy harvester using a magnetic plucking mechanism. *Appl. Energy* **305**, 117838 (2022). 10.1016/j.apenergy.2021.117838

[73] Maamer, B., Boughamoura, A., Fath El-Bab, A. M. R., Francis, L. A. & Tounsi, F. A review on design improvements and techniques for mechanical energy harvesting using piezoelectric and electromagnetic schemes. *Energy Convers. Manag.* **199**, 111973 (2019). 10.1016/j.enconman.2019.111973

[74] Cottone, F., Vocca, H. & Gammaitoni, L. Nonlinear Energy Harvesting. *Phys. Rev. Lett.* **102**, 080601 (2009). 19257728 10.1103/PhysRevLett.102.080601

[75] Cai, M. & Liao, W.-H. Enhanced electromagnetic wrist-worn energy harvester using repulsive magnetic spring. *Mech. Syst. Signal Process.* **150**, 107251 (2021). 10.1016/j.ymssp.2020.107251

[76] Cai, M., Wang, J. & Liao, W.-H. Self-powered smart watch and wristband enabled by embedded generator. *Appl. Energy* **263**, 114682 (2020). 10.1016/j.apenergy.2020.114682

[77] Cai, M. & Liao, W. H. Design, Modeling, and Experiments of Electromagnetic Energy Harvester Embedded in Smart Watch and Wristband as Power Source. *IEEE/ASME Trans. Mechatronics* **26**, 2104 (2021). 10.1109/TMECH.2020.3032536

[78] Vidal, J. V., Carneiro, P. M. R. & Soares dos Santos, M. P. A complete physical 3D model from first principles of vibrational-powered electromagnetic generators. *Appl. Energy* **357**, 122387 (2024). 10.1016/j.apenergy.2023.122387

[79] Ali, A. et al. Advancements in piezoelectric wind energy harvesting: A review. *Results Eng* **21**, 101777 (2024). 10.1016/j.rineng.2024.101777

[80] Li, Y. et al. A novel multi-degree of freedom kinetic energy harvester for self-powered low-power applications in ships. *Energy Convers. Manag.* **302**, 118096 (2024). 10.1016/j.enconman.2024.118096

[81] de Sousa, B. M. et al. Capacitive interdigitated system of high osteoinductive/conductive performance for personalized acting-sensing implants. *NPJ Regen. Med.* **6**, 80 (2021). 34815414 10.1038/s41536-021-00184-6 PMC8611088

[82] Schwartz, G. et al. Flexible polymer transistors with high pressure sensitivity for application in electronic skin and health monitoring. *Nat. Commun.* **4**, 1859 (2013). 23673644 10.1038/ncomms2832

[83] McGlynn, E. et al. The Future of Neuroscience: Flexible and Wireless Implantable Neural Electronics. *Adv. Sci.* **8**, 2002693 (2021). 10.1002/advs.202002693 PMC8132070 34026431

[84] Le, H. T. et al. MEMS inductor fabrication and emerging applications in power electronics and neurotechnologies. *Microsyst. Nanoeng.* **7**, 59 (2021). 34567771 10.1038/s41378-021-00275-w PMC8433479

[85] Bonmassar, G. et al. Microscopic magnetic stimulation of neural tissue. *Nat. Commun.* **3**, 921 (2012). 22735449 10.1038/ncomms1914 PMC3621430

[86] Feng, Y.-Y., Chen, S.-J. & Cheng, S.-P. Development of a miniaturized rotational electromagnetic energy harvester with a liquid metal direct-write process. *Sens. Actuators A: Phys*. **295**, 224 (2019). 10.1016/j.sna.2019.05.001

