# Supplementary material for: Self-adaptive rotational electromagnetic energy generation as an alternative to triboelectric and piezoelectric transductions
Source: Commun Eng. 2024 Jul 31;3:105. doi: 10.1038/s44172-024-00249-6 (PMC11291956; doi:10.1038/s44172-024-00249-6)
Supplement: Supplementary file 1 — Description of Additional Supplementary Files [file 44172_2024_249_MOESM1_ESM.pdf]

Description of Additional Supplementary Files

File name: Supplementary Movie 1

Description: Test scenario using a sliding crank mechanism.

File name: Supplementary Movie 2

Description: Test scenario using a mechanical swaying pendulum.

File name: Supplementary Movie 3

Description: Electromechanical coupling as a function of the rotator angle.
